# Evaluating the global, regional, and national burden of congenital heart disease in infants younger than 1 year: a 1990–2021 systematic analysis for the GBD study 2021

**DOI:** 10.3389/fped.2025.1467914

**Published:** 2025-03-20

**Authors:** Lili Deng, Qinhong Li, Zugen Cheng

**Affiliations:** ^1^ Department of Cardiovascular Medicine, Kunming Children's Hospital, Kunming, Yunnan, China; ^2^ Department of Cardiology, The First Affiliated Hospital of Kunming Medical University, Kunming, Yunnan, China

**Keywords:** congenital heart disease, GBD (global burden of disease), infants, prevalence, mortality

## Abstract

**Background:**

Previous estimates of congenital heart disease (CHD) have been constrained by limited data sources, narrow geographic focus, and a lack of specific assessment of infants younger than 1 year. As part of the Global Burden of Diseases (GBD), Injuries, and Risk Factors Study 2021, this research provides comprehensive estimates of mortality, prevalence, and disability attributable to CHD in infants under 1 year. The study encompasses data from 204 countries and territories, covering the period from 1990 to 2021.

**Methods:**

This cross-sectional analysis utilized data from the 2021 GBD study, encompassing 204 countries and territories. The study focused on infants under 1 year of age with CHD. The GBD dataset was accessed on June 10, 2024.

**Main outcome measures:**

The primary outcomes included prevalence, all-cause, and cause-specific mortality, disability-adjusted life years (DALYs), and the corresponding estimated annual percentage changes (EAPCs). Trends were stratified by region, country, age, and Sociodemographic Index (SDI).

**Results:**

In 2021, CHD resulted in 250,811.32 deaths globally [95% uncertainty interval (UI), 207,821.56–304,084.49], representing a 52.58% decrease from 1990. Among these, 167,985.02 deaths (95% UI, 138,221.77–208,321.59) occurred in infants younger than 1 year. In infants under 1 year old, the 1990 mortality rate for CHD ranked behind neonatal encephalopathy due to birth asphyxia and trauma, diarrheal diseases, neonatal preterm birth, and lower respiratory infections. By 2021, its mortality rates had decreased to the eighth leading cause of death.

**Interpretation:**

CHD remains a significant and rapidly escalating global challenge in child health. While it is difficult to significantly reduce the prevalence of CHD, especially in complex cases, advances in prenatal diagnosis and the availability of medical termination of pregnancy in certain regions have led to demographic changes. Additionally, birth rates, typically lower in high-SDI countries, also influence the prevalence of CHD. Given these factors, the focus should be on improving survival outcomes and quality of life for affected infants. Our findings reveal substantial global disparities in prevalence among infants under 1 year, emphasizing the need for policy reforms that address screening, treatment, and data collection to mitigate these disparities.

## Introduction

Congenital heart disease (CHD), characterized by structural anomalies in the heart and major blood vessels (e.g., the aorta and large veins) present at birth (1). represents the most prevalent congenital malformation worldwide (2). Despite significant advancements in diagnosis and treatment over the past eight decades, CHD remains one of the leading causes of death in children globally. Data from the Metropolitan Atlanta Congenital Defects Program reveal that infants with severe CHD exhibit a significantly lower 1-year survival rate than those with non-severe CHD (75.2% vs. 97.1%). However, the 1 year survival rate for infants with severe CHD has improved over time, rising from 67.4% for those born between 1979 and 1993 to 82.5% for those born between 1994 and 2005 (3). A recent populationbased study of CHD mortality in the United States demonstrated that infants, particularly those with total anomalous pulmonary venous return (TAPVR), exhibit the highest mortality rates (4). A retrospective cohort study on single ventricle CHD found that patients with unbalanced atrioventricular septal defect and hypoplastic left heart syndrome have the lowest five-year survival rates, at 56.1% and 56.7%, respectively. In contrast, patients with pulmonary atresia and double inlet left ventricle have the highest five-year survival rates, at 92.7% and 90.5%, respectively (5). These studies reveal that while substantial advancements have been made in reducing CHD mortality rates in developed regions, such successes have yet to be mirrored in developing areas.

The global under-5 mortality rate has declined significantly since the adoption of the United Nations Sustainable Development Goals (SDGs) in 2015. By 2022, the under-5 mortality rate had fallen to 37 per 1,000 live births, representing a 51% reduction from 2000, and a 14% reduction from 2015. The neonatal mortality rate also decreased to 17 per 1,000 live births, marking a 44% reduction from 2000 (6). Despite these gains, CHD remains responsible for nearly one-third of all major congenital disorders (2). According to the latest study, 80% of CHD patients live at least 35 years (7). Moreover, over 90% of patients who survive their first year of life live to at least 35 years (8). The treatment regimen for infants with CHD encompasses medication therapy, interventional or surgical procedures, and palliative care. While nearly half of these infants require surgical intervention (9), populations in low- and middle-income countries frequently lack timely access to safe, affordable cardiac surgery and rehabilitation services (10). Consequently, these infants are more likely to experience premature death due to cardiopulmonary complications (11).

From 1970 to 2017, the birth prevalence of CHD has increased, peaking at 9.410 cases per 1,000 live births between 2010 and 2017 (12). By 2019, CHD had become the leading cause of non-communicable disease-related deaths among individuals under 20, with 217,000 global deaths attributed to the disease (13). Despite global studies, these primarily offer rough estimates across the entire lifespan, with limited attention to specific age groups, particularly infants under 1 year (14).

The Global Burden of Diseases (GBD), Injuries, and Risk Factors Study—an international research collaboration—systematically produces annual estimates of global mortality and disability. The most up-to-date estimates of prevalence and mortality worldwide were generated in the 2021 update of the GBD. This analysis particularly emphasizes developing data-driven assessments for infants under 1 year. This study integrated a wide range of data sources on mortality and prevalence, applying various statistical techniques to ensure the robustness of the results. In this study, the methods and findings of the GBD 2021 study were detailed, outlining the geographic, temporal, and sociodemographic trends of CHD in infants under 1 year from 1990 to 2021, with specific data drawn from global, regional, and national levels.

## Methods

### Overview and data collection

This cross-sectional study received approval from Kunming Children's Hospital. The ethics committee granted a waiver for informed consent, as the research was limited to data analysis with no personally identifiable information involved. Data for infants younger than 1 year with CHD, including standardized disease definitions and prevalence rates, were sourced using the Global Health Data Exchange query tool developed by the GBD collaborators (15). The tool is available at ghdx.healthdata.org. It is publicly accessible. The 2021 GBD study assessed the prevalence, DALYs, and healthy life expectancy (HALE) for 371 diseases and injuries across 204 countries and territories, including 811 subnational regions, from 1990 to 2021 (15). DALYs are calculated by summing two components: Years of Life Lost (YLL) from premature mortality and Years Lived with Disability (YLD) associated with the health condition. The computational formulas were applied as follows: (1) YLL = Number of deaths × Standard life expectancy at the age of death; (2) YLD = Prevalence of the condition × Disability weight; Where disability weights, derived through systematic expert consensus in the Global Burden of Disease study framework, quantify health loss on a scale from 0 (representing optimal health) to 1 (equivalent to fatal outcomes).

In this study, data were gathered on the number of cases and incidence of CHD, CHDrelated mortality, and CHD-associated DALYs in infants younger than 1 year, along with corresponding rates at global, regional, and national levels. The GBD database does not include data on participants' race and ethnicity, as it does not collect or categorize this information. The mean estimated annual percentage changes (EAPCs) were calculated using linear regression. Alternatively, the World Health Organization's Health Equity Assessment Toolkit (HEAT) is a publicly accessible tool provided by WHO for assessing health inequalities. It is available at who.int/data/gho/health-equity. The toolkit supports customized analysis to evaluate health equity. This study adhered to the Guidelines for Strengthening the Reporting of Observational Studies in Epidemiology (STROBE) (16).

### Sociodemographic Index

The Socio-demographic Index (SDI) measures a country's or region's development level using indicators such as fertility rates, education levels, and per capita income (17). The SDI ranges from 0 to 1, with higher values indicating greater socioeconomic development.

Previous reports have shown that SDI is linked with disease incidence and mortality rates (18).

In this study, countries and regions were categorized into five SDI levels (low, low-middle, middle, high-middle, and high) to investigate the relationship between CHD burden in infants under 1 year and socioeconomic development.

### Statistical analysis

Primary indicators for evaluating the burden of CHD in infants under 1 year encompass prevalence, mortality, and DALYs, accompanied by their respective rates. Each rate was reported per 100,000 population, accompanied by a 95% uncertainty interval (UI) as calculated by the GBD algorithm (19). The dynamics of CHD in infants younger than 1 year were analyzed by calculating the EAPCs to determine the temporal trends in disease burden (20). Linear modeling was used to determine the 95% confidence intervals (CIs) for the EAPCs (21). If both the EAPC and its upper 95% CI are negative, the corresponding rate shows a declining trend. Conversely, if both the EAPC and its lower 95% CI are positive, the corresponding rate exhibits an increasing trend. The relationship between the EAPC, prevalence, mortality, and disability rates of CHD in infants was analyzed using Gaussian curve modeling. The prevalence, incidence, mortality, and DALYs were projected per 100,000 individuals, inclusive of their 95% UI. The analyses and graphical representations were performed using the World Health Organization's Health Equity Assessment Toolkit and R statistical software (Version 3.5.2).

## Result

### CHD in infants: global trends

#### Prevalence

In 2021, the global prevalent cases of CHD in infants younger than 1 year were 1241054.32 (95% UI, 1069339.43–1445303.23). From 1990 to 2021, the global prevalent cases of CHD decreased by 2.9% (95% UI, 1.81%–4.13%). The corresponding prevalence rate experienced an increase from 1000.55 (95% UI, 860.80–1165.48) in 1990 to 979.56 (95% UI, 844.03–1140.78) in 2021 (Table 1). The EAPC was −0.04 (95% CI, −0.07 to −0.02) (Supplementary Table S1). From 1990 to 2021, the prevalence rate of CHD among infants younger than 1 year, irrespective of gender, exhibited a downward trend (Supplementary Table S2).

**Table 1:** Prevalence of congenital heart disease in infants Between 1990 and 2021 at the global and regional level.

Location	1990	Prevalent cases 2021	Percent change, 1990–2021	Prevalence rate		
				1990	2021	Percent change, 1990–2021
Global	1278184.42 (1099651.57–1488870.74)	1241054.32 (1069339.43–1445303.23)	−2.90 (−4.13–1.81)	1000.55 (860.80–1165.48)	979.56 (844.03–1140.78)	−2.10 (−3.33–0.99)
SDI High SDI	98444.34 (87693.25–111046.10)	80440.25 (71092.67–91088.92)	−18.29 (−20.31–16.10)	797.87 (710.74–900.01)	784.03 (692.92–887.82)	−1.73 (−4.16–0.89)
High middle	169901.28 (144448.60–198912.89)	101575.00 (87537.32–118372.54)	−40.22 (−41.91–38.74)	941.90 (800.80–1102.74)	853.28 (735.36–994.39)	−9.41 (−11.98–7.18)
Middle SDI	383924.91 (332003.76–447600.17)	277019.28 (240282.11–320641.93)	−27.85 (−29.58–26.33)	951.92 (823.19–1109.80)	868.31 (753.16–1005.04)	−8.78 (−10.98–6.86)
Low middle	388041.42 (332921.00–453685.43)	391801.06 (336361.90–458432.33)	0.97 (−1.06–3.12)	1063.69 (912.59–1243.63)	1031.50 (885.54–1206.92)	−3.03 (−4.97–0.96)
Low SDI	236955.44 (203207.02–278796.93)	389287.02 (334182.80–457461.97)	64.29 (62.02–66.96)	1158.41 (993.42–1362.96)	1127.01 (967.48–1324.38)	−2.71 (−4.05–1.13)
Regions Andean Latin America	7979.53 (7072.74–9067.54)	8502.35 (7597.55–9692.34)	6.55 (2.10–11.13)	713.25 (632.19–810.50)	695.60 (621.58–792.96)	−2.47 (−6.55–1.72)
Australasia	2133.04 (1807.48–2540.40)	2486.21 (2097.49–2951.40)	16.56 (8.61–25.50)	685.10 (580.54–815.94)	702.68 (592.81–834.15)	2.57 (−4.42–10.43)
Caribbean	6495.31 (5751.86–7390.36)	6398.34 (5628.56–7316.39)	−1.49 (−5.29–2.93)	747.69 (662.11–850.72)	825.60 (726.27–944.06)	10.42 (6.16–15.38)
Central Asia	29827.02 (25173.32–35706.23)	32624.39 (27356.66–39177.92)	9.38 (5.54–13.38)	1556.47 (1313.62–1863.26)	1614.30 (1353.65–1938.58)	3.72 (0.08–7.51)
Central Europe	16621.90 (14204.48–19679.95)	10376.36 (8894.28–12310.45)	−37.57 (−39.62–35.41)	966.53 (825.96–1144.35)	984.86 (844.19–1168.43)	1.90 (−1.45–5.42)
Central Latin America	37601.30 (32872.91–42956.49)	29555.83 (25986.20–33918.53)	−21.40 (−22.73–20.03)	780.89 (682.69–892.11)	763.09 (670.92–875.72)	−2.28 (−3.94–0.58)
Central Sub-SaharanAfrica	29359.80 (24797.27–35454.54)	50237.06 (42797.21–59727.30)	71.11 (61.22–82.88)	1233.59 (1041.89–1489.66)	1169.59 (996.38–1390.54)	−5.19 (−10.67–1.34)
East Asia	239730.35 (202157.97–283695.88)	99515.93 (84946.07–115954.64)	−58.49 (−60.38–56.93)	1032.10 (870.34–1221.38)	833.31 (711.30–970.96)	−19.26 (−22.94–16.23)
Eastern Europe	32269.89 (26689.72–38928.26)	19432.76 (16206.44–23637.61)	−39.78 (−41.12–38.30)	1055.52 (873.00–1273.31)	1069.65 (892.06–1301.10)	1.34 (−0.92–3.83)
Eastern Sub-Saharan Africa	84374.89 (72582.08–98148.20)	128159.52 (110381.07–150254.87)	51.89 (48.60–55.13)	1021.90 (879.07–1188.71)	971.86 (837.04–1139.41)	−4.90 (−6.96–2.87)
High-income Asia Pacific	18805.14 (16517.92–21473.17)	10594.08 (9360.42–11980.82)	−43.66 (−45.17–42.27)	964.55 (847.23–1101.40)	890.02 (786.38–1006.52)	−7.73 (−10.19–5.44)
High-income North America	34739.47 (29963.91–40270.70)	31253.70 (27255.01–36147.27)	−10.03 (−14.90–4.48)	773.27 (666.97–896.39)	777.37 (677.91–899.09)	0.53 (−4.91–6.74)
North Africa and Middle East	95770.07 (84627.36–109202.01)	111369.92 (98215.42–127774.00)	16.29 (14.01–19.01)	912.46 (806.29–1040.43)	941.90 (830.64–1080.63)	3.23 (1.21–5.64)
Oceania	2057.35 (1777.18–2393.80)	4091.98 (3495.87–4773.91)	98.90 (88.01–111.37)	959.61 (828.93–1116.54)	992.36 (847.79–1157.73)	3.41 (−2.25–9.90)
South Asia	351053.66 (299159.05–413135.32)	323474.16 (274973.79–381774.81)	−7.86 (−9.57–5.72)	1084.92 (924.54–1276.79)	1048.83 (891.58–1237.87)	−3.33 (−5.13–1.09)
Southeast Asia	106453.89 (91277.90–124485.64)	95575.97 (81934.18–111964.59)	−10.22 (−11.77–8.76)	896.61 (768.79–1048.49)	863.09 (739.90–1011.09)	−3.74 (−5.40–2.17)
Southern Latin America	7902.27 (6757.52–9299.94)	6047.23 (5323.06–6905.80)	−23.47 (−29.31–18.06)	768.50 (657.17–904.42)	787.30 (693.02–899.08)	2.45 (−5.36–9.69)
Southern Sub-Saharan Africa	15515.64 (13513.40–18240.44)	15790.64 (13738.32–18411.60)	1.77 (−0.74–4.54)	1001.66 (872.40–1177.57)	992.31 (863.34–1157.01)	−0.93 (−3.38–1.76)
Tropical Latin America	24705.75 (21731.08–28187.90)	24970.90 (21945.23–28361.32)	1.07 (−2.37–4.64)	757.16 (666.00–863.88)	731.28 (642.67–830.56)	−3.42 (−6.71–0.01)
Western Europe	35141.05 (32486.09–37955.18)	32396.94 (28997.91–35990.16)	−7.81 (−11.81–3.31)	767.20 (709.24–828.64)	793.54 (710.28–881.55)	3.43 (−1.06–8.48)
Western Sub-Saharan Africa	99647.09 (84719.78–117718.31)	198200.04 (168604.32–232263.18)	98.90 (95.69–102.71)	1204.53 (1024.09–1422.98)	1170.32 (995.57–1371.46)	−2.84 (−4.41–0.98)

Data in parentheses are 95% uncertainty intervals. SDI, Socio-demographic Index.

#### Mortality

**Figure 1:**
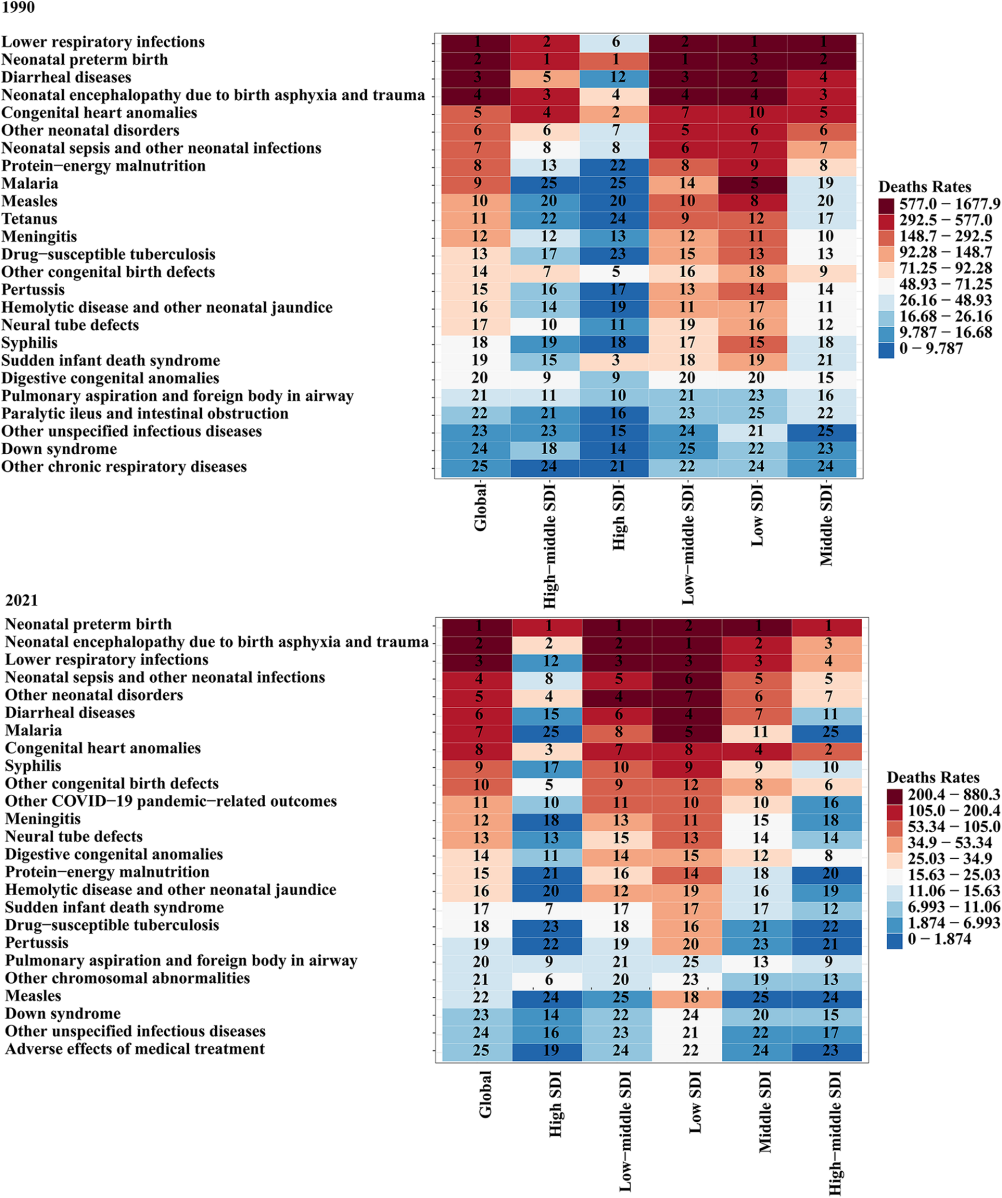
Leading causes of death in infants younger than 1 year. SDI, Socio-demographic Index.

Over the past three decades, the global number of CHD-associated deaths in infants decreased by 54.58% (369851.15;95% UI, 218275.80–472876.34) in 1990 vs. 167985.02 (95% UI, 138221.77–208321.59) in 2021. Similarly, the CHD-associated death rate decreased from 289.52 (95% UI, 170.86–370.16) per 100,000 in 1990 to 132.59 (95% UI, 109.10–164.43) per 100,000 in 2021 (Table 2); The EAPC was −2.35 (95% CI, −2.42 to 2.28) (Supplementary Table S3). Globally, in 2021, the top five causes of infant mortality were neonatal preterm birth, neonatal encephalopathy due to birth asphyxia and trauma, lower respiratory infections, neonatal sepsis and other neonatal infections, and other neonatal disorders. By contrast, in 1990, the predominant causes included preterm birth, lower respiratory infections, diarrheal diseases, congenital heart anomalies, and neonatal encephalopathy due to birth asphyxia and trauma (Figure 1).

**Table 2:** Mortality of congenital heart disease in infants Between 1990 and 2021 at the global and regional level.

Location	1990	Death cases (all ages) 2021	Percent change	1990	Deaths cases (<1 year) 2021	Percent change	Mortality (<1 year)		
							1990	2021	Percent change
Global	528947.97 (310319.52–679086.63)	250811.32 (207821.56–304084.49)	−52.58 (−63.16–−20.09)	369851.15 (218275.80–472876.34)	167985.02 (138221.77–208321.59)	−54.58 (−65.22–−23.77	289.52 (170.86–370.16)	132.59 (109.10–164.43)	−54.20 (−64.93–−23.14)
SDI High SDI	24348.18 (21239.64–26304.68)	7257.10 (6429.65–8463.61)	−70.19 (−74.19–61.59)	14843.30 (12784.14–16211.09)	3083.55 (2455.09–3785.76)	−79.23 (−83.69–73.11)	120.30 (103.61–131.39)	30.05 (23.93–36.90)	−75.02 (−80.39–67.66)
High middle	81058.96 (57648.72–99495.94)	16289.13 (13822.95–18917.53)	−79.90 (−84.71–69.06)	53142.14 (37657.07–65578.07)	8361.79 (6750.88–10096.36)	−84.27 (−88.55–74.79)	294.61 (208.76–363.55)	70.24 (56.71–84.81)	−76.16 (−82.64–61.80)
Middle SDI	172912.04 (109843.63–228439.80)	55120.67 (46568.49–66284.92)	−68.12 (−76.60–42.12)	119531.19 (74338.79–157377.80)	34685.93 (28450.89–42838.63)	−70.98 (−79.04–43.88)	296.37 (184.32–390.21)	108.72 (89.18–134.28)	−63.32 (−73.51–29.06)
Low middle	157723.39 (84745.36–213746.76)	81206.75 (64189.73–100979.42)	−48.51 (−62.67–9.30)	116566.37 (66173.98–154077.31)	58517.51 (45420.41–73970.21)	−49.80 (−63.72–1.91)	319.53 (181.39–422.35)	154.06 (119.58–194.74)	−51.79 (−65.16–2.13)
Low SDI	92466.81 (34131.42–134904.55)	90668.03 (64963.50–122202.71)	−1.95 (−25.70–98.70)	65452.89 (25782.10–98502.63)	63146.26 (44471.56–86111.54)	−3.52 (−27.39–77.62)	319.98 (126.04–481.55)	182.81 (128.75–249.30)	−42.87 (−57.00–5.18)
Regions Andean Latin America	5293.58 (2890.32–6873.14)	2474.08 (1881.44–3166.79)	−53.26 (−67.93–6.13)	3853.56 (2150.35–5050.23)	1741.61 (1279.50–2256.37)	−54.81 (−69.92–7.97)	344.45 (192.21–451.41)	142.49 (104.68–184.60)	−58.63 (−72.47–15.77)
Australasia	349.21 (325.34–381.34)	184.03 (154.27–215.51)	−47.30 (−57.25–37.36)	194.19 (176.44–217.50)	77.78 (55.52–99.50)	−59.95 (−71.62–48.64)	62.37 (56.67–69.86)	21.98 (15.69–28.12)	−64.76 (−75.02–54.80)
Caribbean	4107.51 (3104.77–5089.43)	2631.68 (1646.92–4486.08)	−35.93 (−55.86–7.83)	2803.50 (2204.41–3445.11)	1820.22 (1190.29–2766.82)	−35.07 (−54.96–4.43)	322.72 (253.76–396.58)	234.87 (153.59–357.01)	−27.22 (−49.52–17.05)
Central Asia	4237.08 (3679.02–4788.23)	4629.19 (3672.82–5682.95)	9.25 (−10.68–33.25)	3083.46 (2678.82–3483.85)	3235.58 (2518.38–4052.93)	4.93 (−16.64–30.52)	160.90 (139.79–181.80)	160.10 (124.61–200.54)	−0.50 (−20.96–23.76)
Central Europe	5307.42 (4590.71–5862.28)	918.01 (762.62–1056.65)	−82.70 (−86.69–79.25)	3863.71 (3277.66–4317.72)	533.89 (426.65–638.72)	−86.18 (−89.88–82.82)	224.67 (190.59–251.07)	50.67 (40.50–60.62)	−77.44 (−83.48–71.95)
Central LatinAmerica	12271.30 (10828.60–13915.13)	8827.75 (6992.31–11126.92)	−28.06 (−43.78–7.38)	9107.01 (7971.96–10461.55)	5972.96 (4481.24–7729.11)	−34.41 (−52.08–12.04)	189.13 (165.56–217.26)	154.21 (115.70–199.55)	−18.46 (−40.43–9.35)
Central Sub-Saharan Africa	7800.71 (2444.84–14236.75)	6539.90 (4109.71–10622.07)	−16.16 (−40.52–101.21)	5831.35 (2007.55–10856.06)	4610.33 (2863.27–7690.21)	−20.94 (−44.13–72.88)	245.01 (84.35–456.13)	107.34 (66.66–179.04)	−56.19 (−69.04–4.21)
East Asia	127881.84 (82116.96–176228.71)	20837.54 (16839.94–25796.87)	−83.71 (−88.75–71.11)	77203.28 (47758.74–109065.77)	9599.74 (7230.75–12677.99)	−87.57 (−92.05–76.07)	332.38 (205.61–469.55)	80.38 (60.55–106.16)	−75.82 (−84.55–53.45)
Eastern Europe	8288.46 (7425.40–9759.60)	2050.66 (1813.72–2417.73)	−75.26 (−80.43–69.53)	5571.94 (4939.25–6605.19)	889.18 (690.68–1088.03)	−84.04 (−88.78–79.15)	182.25 (161.56–216.05)	48.94 (38.02–59.89)	−73.15 (−81.11–64.92)
Eastern Sub-Saharan Africa	28862.20 (8249.32–55988.88)	23552.56 (14987.91–41886.87)	−18.40 (−44.04–110.36)	21032.51 (6215.60–41791.66)	16569.97 (10205.74–29707.48)	−21.22 (−45.04–84.31)	254.73 (75.28–506.16)	125.65 (77.39–225.28)	−50.67 (−65.59–15.40)
High-income Asia Pacific	4302.19 (3538.18–4794.72)	760.16 (618.26–963.96)	−82.33 (−84.90–73.82)	2440.46 (1935.01–2813.76)	259.08 (196.31–355.19)	−89.38 (−91.59–82.58)	125.18 (99.25–144.32)	21.77 (16.49–29.84)	−82.61 (−86.22–71.47)
High-income North America	6645.48 (5828.76–7180.40)	2875.46 (2616.92–3530.58)	−56.73 (−61.74–42.02)	3829.50 (3269.42–4203.74)	1240.58 (1021.29–1553.11)	−67.60 (−74.34–56.33)	85.24 (72.77–93.57)	30.86 (25.40–38.63)	−63.80 (−71.33–51.21)
North Africa and Middle East	95835.21 (42899.80–135845.83)	35272.23 (28067.26–43669.89)	−63.19 (−73.62–28.15)	76903.10 (34741.63–107155.17)	26430.26 (20940.49–33127.98)	−65.63 (−75.14–34.47)	732.70 (331.00–1020.93)	223.53 (177.10–280.18)	−69.49 (−77.93–41.84)
Oceania	1034.72 (389.98–1525.92)	1751.69 (805.98–2616.61)	69.29 (30.07–132.88)	749.45 (246.98–1114.79)	1283.68 (493.77–1955.23)	71.28 (29.09–141.28)	349.57 (115.20–519.97)	311.31 (119.75–474.17)	−10.94 (−32.88–25.45)
South Asia	115427.38 (72640.39–154224.07)	57259.61 (41722.85–78496.76)	−50.39 (−65.69–4.76)	83891.13 (56739.96–111278.27)	41537.85 (28718.90–60500.72)	−50.49 (−66.32–0.01)	259.26 (175.35–343.90)	134.68 (93.12–196.17)	−48.05 (−64.67–4.93)
Southeast Asia	46651.51 (23423.77–62790.49)	23227.32 (19107.13–28529.57)	−50.21 (−63.30–2.68)	33763.40 (16423.17–45787.06)	16181.75 (13077.88–20228.95)	−52.07 (−64.64–0.35)	284.37 (138.33–385.64)	146.13 (118.10–182.68)	−48.61 (−62.08–7.59)
Southern Latin America	2200.34 (1849.42–2569.95)	1044.16 (878.05–1247.23)	−52.55 (−62.81–40.19)	1702.84 (1407.74–2049.03)	688.66 (542.36–867.25)	−59.56 (−69.88–46.89)	165.60 (136.90–199.27)	89.66 (70.61–112.91)	−45.86 (−59.67–28.90)
Southern Sub-Saharan Africa	1942.88 (1576.46–2514.26)	1713.00 (1158.66–2268.83)	−11.83 (−36.57–24.79)	1413.62 (1143.25–1852.02)	1147.61 (772.91–1626.39)	−18.82 (−44.23–16.22)	91.26 (73.81–119.56)	72.12 (48.57–102.21)	−20.98 (−45.72–13.13)
Tropical Latin America	8532.92 (7311.92–9829.50)	5314.98 (4359.96–6380.11)	−37.71 (−52.17–20.73)	6448.42 (5405.26–7569.21)	3929.92 (3101.27–4832.81)	−39.06 (−54.83–19.39)	197.63 (165.66–231.98)	115.09 (90.82–141.53)	−41.76 (−56.84–22.97)
Western Europe	8154.53 (7096.84–8796.13)	2426.00 (2128.62–2835.11)	−70.25 (−74.69–62.84)	4774.65 (4108.61–5244.21)	1050.07 (810.98–1289.36)	−78.01 (−83.55–71.17)	104.24 (89.70–114.49)	25.72 (19.86–31.58)	−75.33 (−81.55–67.66)
Western Sub-Saharan Africa	33821.50 (9343.84–51421.34)	46521.31 (28523.61–65065.35)	37.55 (4.78–226.63)	21390.10 (6311.29–30465.34)	29184.33 (17446.30–39402.27)	36.44 (3.96–197.85)	258.56 (76.29–368.26)	172.33 (103.02–232.66)	−33.35 (−49.22–45.49)

Data in parentheses are 95% uncertainty intervals. SDI, socio-demographic Index.

#### Disability-adjusted life years

In 2019, the global number of CHD- associated DALYs in infants under 1 year decreased by 54.45% from 1990 to 2021 (33278578.86; 95% UI, 19684030.06–42527804.16) in 1990 vs. 15158581.33 (95% UI, 12507441.29–18793629.80) in 2021 (Supplementary Table S4 in Supplementary Material); The EAPC was −2.34 (95% CI, −2.41 to −2.27) (Supplementary Table S5).

### CHD in infants: SDI regional trends

#### Prevalence

In 2021, the low SDI region had the highest number of CHD cases. (1127.01; 95% UI, 967.48–1324.38). The prevalent cases in the low SDI region increased by 64.29% (95% UI, 62.02–66.96) (Table 1). The EAPC in the low SDI region was −0.06 (95% CI, −0.08 to 0.05). The high-middle SDI region experienced the largest decline in the prevalence of CHD among infants, with an EAPC of −0.28 (95% CI, −0.33 to −0.23) (Supplementary Table S5).

#### Mortality

In all five SDI regions, the mortality rate associated with CHD exhibited a decline. The high-middle SDI region had the greatest decrease (76.16%) in CHD-associated mortality; the low SDI region had the lowest decrease (42.87%) in CHD-associated mortality; However, the highest SDI region had the lowest number of CHD-associated deaths in 2021 (3083.55; 95% UI, 2455.09–3785.76) (Table 2). The high-middle SDI region had the lowest EAPC in infants with CHD-associated mortality rate (−5.09; 95% CI, −5.34 to −4.85) (Supplementary Table S3).

#### Disability-adjusted life years

In 2021, the low SDI region had the highest number of CHD-associated DALYs (5689689.21;95% UI, 4016523.89–7743144.24) with a decrease of 3.38% from 1990 to 2021; The EAPC was −1.56 (95% CI, −1.65 to −1.47) (Supplementary Table S4). The highmiddle SDI region exhibited the most substantial decline, with an 84.15% reduction in the number of DALYs attributed to CHD (Supplementary Table S4); The EAPC was −5.07 (95%CI, −5.31 to −4.83) (Supplementary Table S5).

### CHD in infants: geographic regional trends

#### Prevalence

Among the 21 geographic regions, South Asia had the highest CHD cases in 2021, totaling 323,474.16 (95% UI, 274,973.79–381,774.81). whereas Australasia had the fewest cases, with 2,486.21 (95% UI, 2,097.49–2,951.40). The prevalence of CHD peaked in Central Asia, registering at 1614.30 per 100,000 infants (95% UI, 1353.65–1938.58). Over the period from 1990 to 2021, the prevalence of CHD experienced an increase of 3.72% (95% UI, 0.08%–7.51%). In contrast, Andean Latin America exhibited the lowest prevalence of CHD, with a rate of 695.60 per 100,000 infants (95% UI, 621.58–792.96). Between 1990 and 2021, the prevalence of CHD in this region declined by 2.47% (95% UI, −6.55%–1.72%) (Table 1). From 1990 to 2021, the Caribbean had the largest increase in the prevalence of CHD (EAPC, 0.31; 95% CI, 0.29–0.33), whereas Central Asia had the smallest increase (EAPC,0.02; 95% CI, −0.01–0.04) (Supplementary Table S1 in Supplementary Material). In 2021, Central Asia (SDI, 0.68) had the highest prevalence of CHD, whereas Andean Latin America (SDI, 0.65) had the lowest prevalence (Supplementary Table S6 and Table 1).

#### Mortality

In 2021, South Asia had the highest number of CHD-associated deaths, totaling 41,537.85 (95% UI, 28,718.90–60,500.72), representing a 50.49% decrease from 83,891.13 deaths (95% UI, 56,739.96–111,278.27) in 1990. Oceania had the highest CHD-associated mortality rate (311.31; 95% UI, 119.75–474.17), a 10.49% decrease from 349.57 (95% UI, 115.20–519.97) in 1990 (Table 2). Oceania had the smallest decrease in CHD-associated mortality rate (EAPC, −0.26; 95% CI, −0.40 to −0.12), whereas East Asia had the largest decrease (EAPC, −5.38; 95% CI, −5.74 to −5.02) (Supplementary Table S3).

#### Disability-adjusted life years

In 2021, South Asia had the highest number of CHD-associated DALYs (3751572.08; 95% UI, 2592766.38–5449500.61), whereas Australasia had the lowest number (7156.84;95% UI, 5151.54–9089.08). Oceania had the highest DALYs rate (27994.29;95% UI, 10819.0842605.12); High-income Asia Pacific had the lowest DALYs rate (2010.74;95% UI, 1540.38–2738.21). From 1990 to 2021, Central Asia had the largest increase in the DALYs rate (EAPC, 0.24; 95% CI, −0.12 to 0.60); East Asia had the largest decrease (EAPC, −5.36; 95% CI, −5.71 to −5.00). The global SDI was 0.67 in 2021; 9 regions (e.g., High-income Asia Pacific) had rates of DALYs that were higher than the global mean, whereas 12 regions (e.g., Oceania) had rates that were lower than the global mean (11964.671) (Supplementary Table S6).

### CHD in infants younger than 1 year: national trends

#### Prevalence

**Figure 2:**
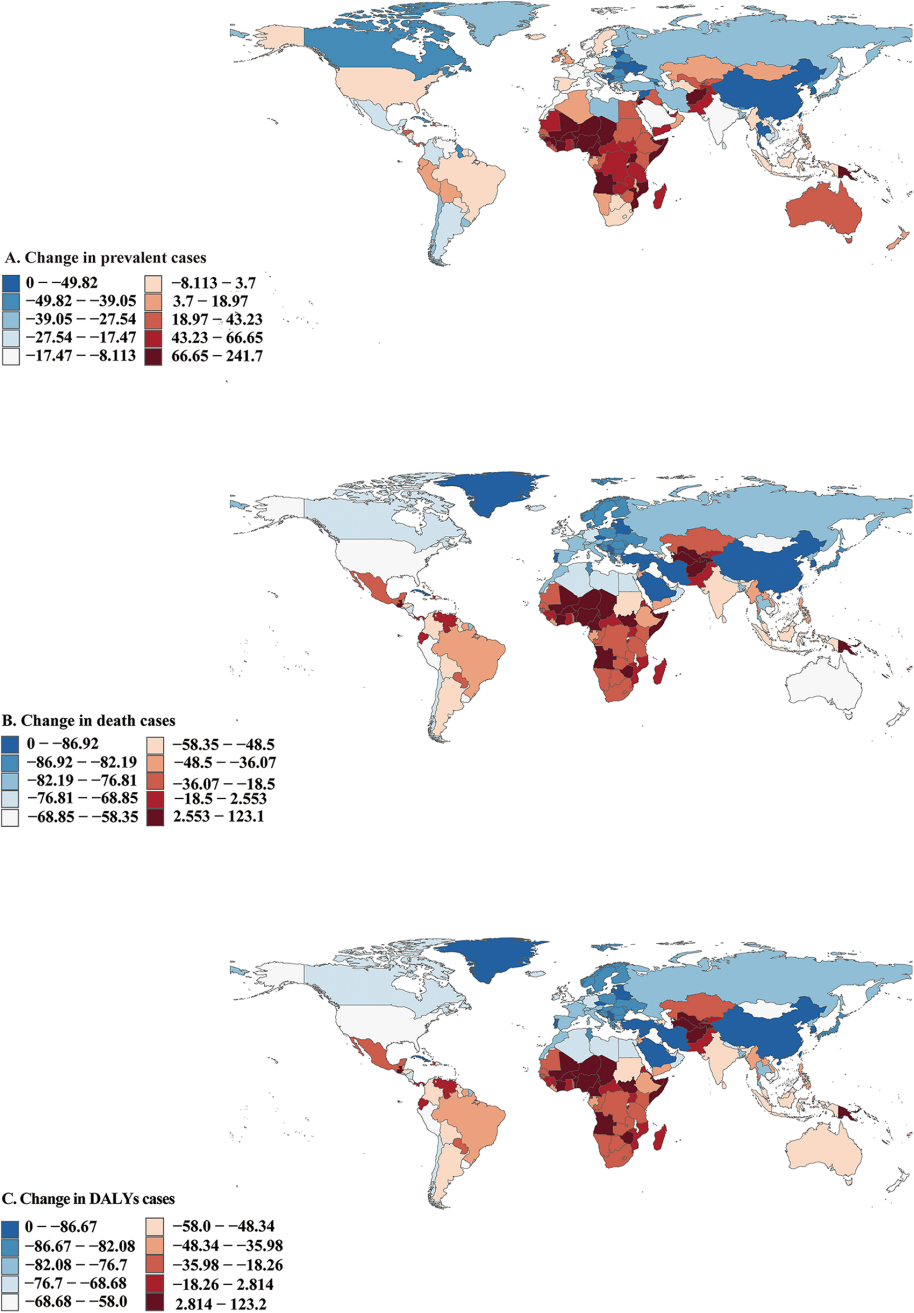
Prevalent, death, and disability-adjusted life-years (DALYs) cases of congenital heart disease in 204 countries and territories. **(A)** Prevalent cases. **(B)** Death cases. **(C)** DALYs cases.

In 2021, among 204 countries, India had the most cases of CHD (221053.67; 95% UI, 188632.73–261811.24). a 13·7% decrease from 256148.89 (95% UI, 218504.15–301463.11) prevalent cases in 1990 (Figure 2A and Supplementary Figure S1); Tajikistan had the highest prevalence rate of CHD (1836.53; 95% UI, 1532.60–2218.51) (Supplementary Figure S8); Croatia (EAPC, 0.93; 95% CI, 0.72–1.13) had the largest increases in prevalence. Canada (EAPC, −1.74; 95% CI, −1.94 to −1.54) had the largest decreases (Supplementary Figure S4A and Supplementary Table S7). In 2021, Tajikistan (SDI, 0.54) had the highest prevalence of CHD, whereas Puerto Rico (SDI, 0.83) had the lowest prevalence (Supplementary Figure S2 and Supplementary Table S6). The global prevalence of CHD in 2021 was 979.56 (95% UI, 844.03–1140.78); The prevalence of CHD was above the global mean in 71 countries and below it in 133 countries (Supplementary Figure S2).

#### Mortality

In 2021, India had the highest number of CHD-associated deaths (29775.10; 95% UI, 20947.27–43450.45), a 49.05% decrease from 58442.58 (95% UI, 37628.97–79807.86) deaths cases in 1990 (Figure 2B and Supplementary Figure S3). Afghanistan (611.85; 95% UI, 310.24–874.40) had the highest CHD-associated mortality rate, a 53.24% decrease from 1308.62 (95% UI, 328.09–2099.78) mortality rate in 1990; Monaco (4.47;95% UI, 1.96–8.69) had the lowest mortality rate (Supplementary Figures S9 and S10). Guatemala (EAPC, 4.43; 95% CI, 3.57–5.29) had the greatest increases in the mortality rate; Belarus (EAPC, −8.45; 95% CI, −9.55 to −7.33) had the greatest decreases (Supplementary Figure S4B and Supplementary Table S7). Afghanistan (SDI, 0.34) had the highest CHD-associated mortality rate, whereas Monaco (SDI, 0.91) had the lowest mortality rate. The global CHD-associated mortality rate in 2021 was 132.59 (95% UI, 109.10–164.43). The rates were above the global mean in 51 countries and below the global mean in 153 countries (Supplementary Figure S11).

#### Disability-adjusted life years

In 2021, India had the highest number of CHD-associated infants DALYs (2688959.44; 95% UI, 1897797.07–3912767.96), a 48.94% decrease from 5265978.06 (95% UI, 3394754.07–7185888.72) DALYs cases in 1990 (Figure 2C and Supplementary Figure S12). Afghanistan had the highest rate of infants' CHD-associated DALYs (54984.35; 95% UI, 27920.56–78549.77), a 53.23% decrease from 117568.83 (95% UI, 29547.93–188561.97) DALYs rate in 1990 (Supplementary Figures S6 and S7). Guatemala (EAPC, 4.4;95% UI, 3.55–5.26) had the greatest increase in DALYs rate. Saudi Arabia (EAPC, −8.02; 95% CI, 8.10 to −7.94) and Belarus (EAPC,−8.38; 95% CI, −9.47 to −7.27) had the greatest decreases (Supplementary Figure S4C and Supplementary Table S7). Afghanistan (SDI,0.34) had the highest rate of infants’ CHD-associated DALYs; San Marino (SDI, 0.89) had the lowest rate (Supplementary Figures S6 and S13).

#### Factors influencing EAPCs

**Figure 3:**
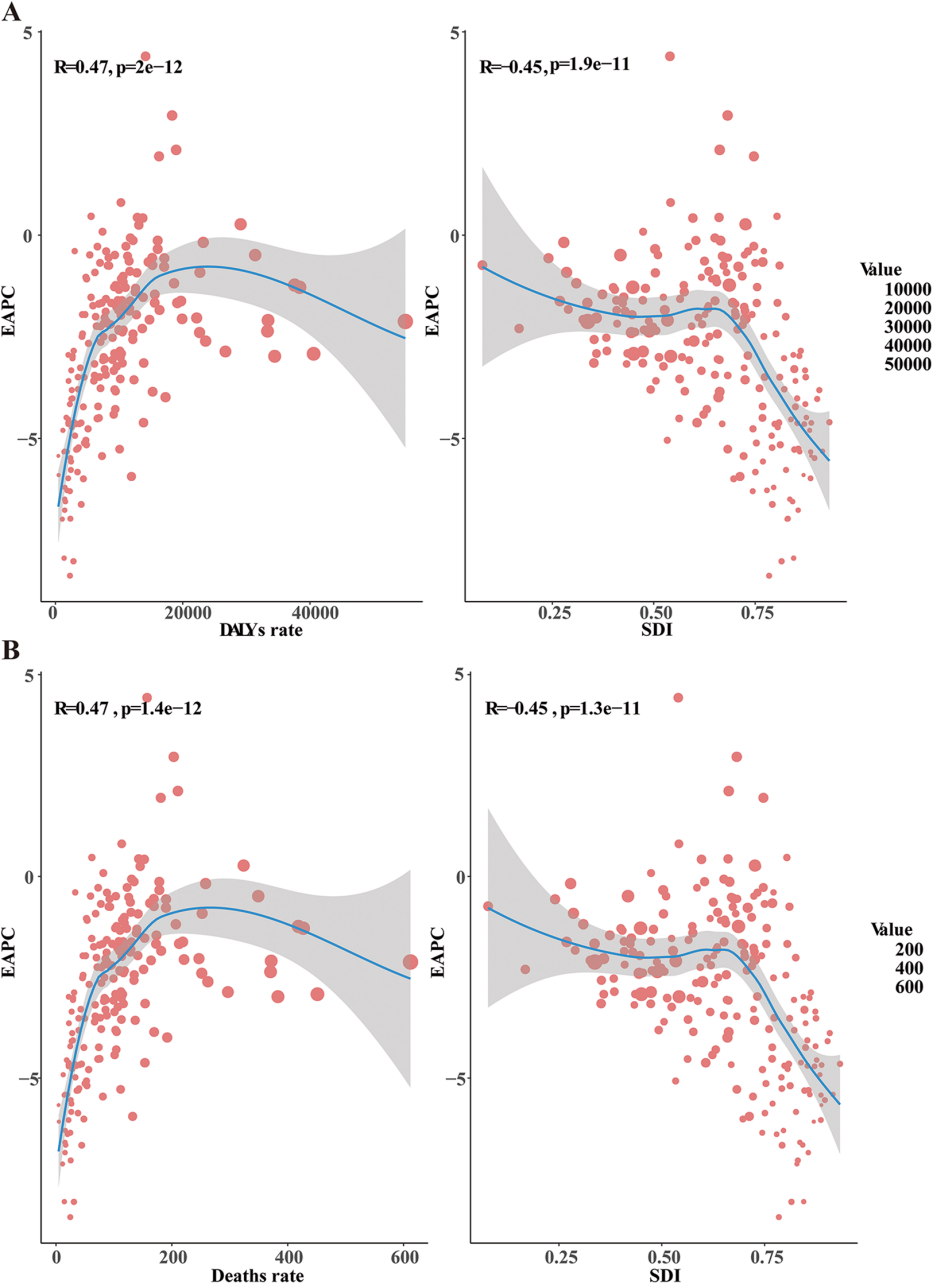
Factors influencing EAPC. EAPC, estimated annual percentage change; DALYs, disability-adjusted life-years; SDI, sociodemographic index. **(A)** The relationship between EAPC and both the DALYs rate and SDI **(B)**, the relationship between EAPC and both the death rate and SDI.

Significant differences in the EAPC were observed compared to the prevalence, mortality rate, and number of DALYs in 1990 and the Socio-Demographic Index (SDI) in 2021. The prevalence rates in 1990 represent the baseline disease burden, while the SDI can be considered an indicator of the level of medical care. The outcome of this study revealed a significant negative correlation between EAPCs and DALYs rates (Pearson *r* = −0.45, *p* < 0.001), indicating that as EAPCs increase, DALYs rates tend to decrease (Figure 3A). Additionally, the EAPC in prevalence was positively correlated with SDI, while the EAPC in mortality rate was negatively correlated with SDI (Pearson *r* = −0.45, *p* < 0.001), underscoring the influence of healthcare quality on changes in disease burden (Figure 3B).

## Discussion

In the realm of pediatric healthcare, CHD in infants younger than 1 year has garnered significant attention worldwide. However, it remains inadequately addressed within public health and epidemiological research. Our comprehensive study underscores a notable decline in the global burden of CHD in infants over the past three decades, as evidenced by variations in prevalence, mortality rates, and DALYs. From 1990 to 2021, the number of CHD cases in infants under 1 year decreased from 1,278,184.42 to 1,241,054.32. This decline reveals regional disparities, socio-populational differences, and age-structural heterogeneities. Remarkably, there was a 2.9% reduction in CHD cases among infants, a dramatic 54.20% drop in mortality rates, and a significant 54.07% decrease in DALYs over this period. Although the prevalence of CHD among infants has not markedly declined on a global scale, the substantial reductions in mortality rates and DALYs suggest a continuous decrease in the disease burden worldwide. This trend reflects advancements in medical interventions, improved healthcare access, and heightened awareness, all contributing to better outcomes for infants afflicted with CHD. Technological evolution has enabled increasingly frequent diagnoses of CHD, including those that are simple and often asymptomatic, which may make these conditions appear more prevalent. On the other hand, the possibility of prenatal diagnosis and the medical termination of pregnancies involving fetuses with complex heart diseases has affected both the prevalence of CHD and its associated mortality and comorbidities. Further research is essential to address the persisting disparities and develop targeted public health strategies to sustain and enhance these positive trends globally.

The significance of CHD as a cause of childhood mortality has been rising. From 1990 to 2021, high SDI countries experienced a notable decline in the proportion of childhood deaths due to CHD. Conversely, while respiratory illnesses remain among the top three causes of death in low SDI countries, their rank has significantly declined in high SDI nations. This pattern exemplifies the epidemiological transition observed in nations with advancing economies and public health infrastructures, where the disease burden shifts from infectious to non-communicable diseases. The observed decline in CHD-related mortality in high SDI regions underscores the impact of enhanced healthcare availability, early diagnosis, and advanced treatment options. While improved diagnostic capacity is gradually emerging in low-SDI settings, critical disparities persist in care access and outcomes. Children with complex CHD in high-SDI countries not only benefit from superior survival rates but also face extended lifespans requiring sustained medical management—a phenomenon less prevalent in resource-limited regions where mortality often occurs earlier in the disease course. These outcomes paradoxically amplify the longterm care burden in high-SDI settings, where surgical interventions, advanced technologies, and multidisciplinary follow-up impose exceptional financial and logistical demands on healthcare systems.

Three demographic realities further complicate global CHD epidemiology: declining birth rates in high-SDI countries, increasing prenatal detection of severe cardiac anomalies, and selective pregnancy terminations for complex CHD diagnoses. These intersecting factors create an epidemiological paradox where regions with the greatest treatment capacity paradoxically demonstrate the reduced prevalence of severe cases, while low-SDI regions face rising CHD burdens compounded by diagnostic delays and constrained resources. This evolving landscape demands urgent policy attention, emphasizing the need for contextspecific strategies that address both technological innovation in high-resource settings and foundational healthcare strengthening in developing regions. Sustained investment must prioritize equitable resource allocation, workforce training, and ethical frameworks for prenatal care—essential steps toward mitigating CHD's global health impact.

Precisely estimating the mortality and morbidity associated with CHD has consistently been challenged by various complexities. While the GBD 2021 mortality estimates align with previous research findings (14), designating CHD as the primary cause of death may lead to an underestimation of the overall disease burden. The frequent association of CHD with other conditions, such as pulmonary complications, genetic syndromes, and cardiac anomalies, leads to this underestimation (22). Furthermore, the spontaneous remission of certain types of CHD, such as ventricular septal defects and atrial septal defects, complicates the estimation of prevalence. As with many diseases, data collection and reporting strategies for CHD exhibit significant variations across regions, countries, and hospitals. Variations across regions, countries, and hospitals contribute to the substantial variability in CHD data accuracy and comprehensiveness. Factors such as differing coding systems, the inclusion of multiple CHD, and the consideration of pregnancy terminations all play a role. The ICD-9 Clinical Modification coding system may exhibit low sensitivity and high false-positive rates for certain CHDs when compared to clinical nomenclature (23). This discrepancy underscores the ongoing challenges in achieving uniformity and precision in disease classification and reporting. Moreover, ongoing debate surrounds the so-called “unnatural history” of individuals who have undergone straightforward repairs of ventricular and atrial septal defects during childhood. The central question is whether these individuals should still be considered as living with CHD (24). This issue is further complicated for lesions that lack hemodynamic consequences, such as patent ductus arteriosus and certain types of pulmonary stenosis, which are less likely to be clinically monitored, thereby leaving their remission rates largely unknown. While individuals with moderate to large septal defects in the ventricles or atria often experience significant health risks if not treated, those with minor ventricular septal defects, partially closed atrial septal defects, or patent foramen ovale generally do not show increased morbidity or mortality from these conditions. Among CHD, ventricular septal defects are the most common, with at least 25% resolving on their own (25). Some studies report closure rates as high as 20% per year, though these rates vary significantly across studies due to differing methodologies (6, 26, 27).

Previous studies have utilized diverse methodologies to tackle the aforementioned challenges. In 2010, the US Centers for Disease Control and Prevention (CDC) showed that the prevalence of CHD in children and adolescents ranged from 1 in 250 to 1 in 59. This estimate was derived from harmonizing CHD definitions and variable selections across administrative data sources from three locations in the US (Massachusetts, New York, and the European Union), marking the first population-based CHD surveillance among US adolescents. Additionally, between 2011 and 2013, 1 in 157 children aged 1–10 years and 1 in 680 adolescents and adults aged 11–64 years had a heart defect noted in their health records during a medical visit (28). However, to date, such estimates in the US have largely relied on Canadian data or extrapolations from Canadian data to the US population (29). The prevalence estimated based on Canadian data may not accurately reflect the true prevalence in the US due to differences in sociodemographic characteristics and healthcare accessibility and utilization. A meticulous analysis of the global prevalence of structural heart disease reveals that the incidence of CHD is remarkably consistent worldwide (30). However, it is important to note that the most common defect, ventricular septal defect (VSD), was not included in their calculations (30). This omission underscores the need for more comprehensive data collection to accurately reflect the true burden of CHD globally. A gradual increase in the global birth incidence of CHD from 1970 to 2017 was reported (12). This rise is primarily attributed to the increased incidence of “mild” CHD lesions, such as VSD, atrial septal defects, and patent ductus arteriosus. The observed trend is likely due to improvements in postnatal diagnostic capabilities, which have enhanced the detection and reporting of these conditions (12).

The global health landscape, especially regarding children's health, is constantly changing. Reliable and regularly updated information on disease impact is essential for monitoring these shifts and fostering sustainable strategies that provide high-standard care for all children requiring it. The GBD estimates for CHD offer invaluable insights into global health, providing a clearer roadmap for the efficient use and allocation of resources, especially in cardiovascular and non-communicable disease care. This is particularly important for low- and middle-income countries, where progress in treating CHD has been relatively stagnant. The United Nations has prioritized reducing premature deaths caused by non-communicable diseases, including acquired and congenital heart diseases (31). However, to meet the specific Sustainable Development Goal of eliminating preventable deaths among newborns and children under five, policymakers must establish robust accountability measures, eliminate obstacles, and enhance access to pediatric cardiology services. In addition to policy efforts, there is a need to enhance public health education to raise awareness about CHD and the importance of early screening. Further research and development must be promoted to enhance the accessibility and effectiveness of diagnostic technologies and treatment methods. Through international cooperation and resource sharing, the management and treatment of congenital heart disease (CHD) can be improved globally, particularly in regions with limited medical resources. Furthermore, establishing a global monitoring and reporting system for CHD can better track disease trends and treatment outcomes, enabling policymakers to make more informed decisions based on real-time data, ensuring that every child receives timely and effective treatment. Only through a collective effort from society can the burden of CHD be truly reduced, creating a healthier future for all children. Notably, accurate data on disease burden is key to improving global children's health. Through effective policies, public education, research and development, and international cooperation, better medical services and quality of life can be provided for all children in need.

Several healthcare policies and interventions could be crucial to address the observed trends in CHD. First, in high-SDI regions, advanced medical technologies, such as prenatal screening, early diagnosis, and surgical interventions, have significantly improved the management and treatment of CHD. Policies that ensure universal access to high-quality healthcare services contribute to better detection and treatment of CHD, thereby increasing the survival rates of children born with the condition. In contrast, low-SDI regions often face numerous challenges, such as limited access to healthcare facilities, specialized medical professionals, and life-saving surgeries. Interventions from international aid, partnerships, and non-governmental organizations (NGOs) may offer some assistance, but significant barriers to accessing basic healthcare services—including prenatal care and diagnostic tools—persist. These barriers severely hinder efforts to reduce CHD mortality in these regions. Moreover, in high-SDI countries, public health policies focusing on maternal and child health often include specialized care for congenital conditions. Policies that support early screening programs, such as newborn cardiac defect screening, play a crucial role in identifying CHD before symptoms appear, allowing for timely intervention. Low-SDI countries, however, often lack such policies, leading to delayed diagnoses and an increase in severe cases requiring intensive treatment. Governments in these regions may prioritize infectious diseases and maternal mortality over congenital conditions, which can result in lower awareness and fewer resources allocated for the prevention and treatment of CHD. Lastly, in high-SDI countries, comprehensive health systems and funding mechanisms support ongoing research into CHD, aiding the development of better treatments and interventions. High-income regions often benefit from cutting-edge research, treatment innovations, and long-term follow-up programs for children with CHD. On the other hand, international health organizations and charitable initiatives are often the main drivers of progress in low-income regions. While these initiatives can be incredibly valuable, their reach and impact may be limited without sustained local government support and the development of permanent infrastructure for CHD care.

In summary, as the survival rate of CHD patients has improved, they face various complications, lifestyle issues, and challenges for special populations in adulthood, and further optimization of medical services and social support is needed (32).

## Limitations

Several limitations affect this study. One major constraint is that this analysis relies on the GBD framework, which utilizes aggregated secondary data sources instead of original primary datasets. The availability of national registry data constrains the accuracy of the database, the significant number of undiagnosed CHD cases in children under 1 year, and the lack of information on other risk factors related to CHD. A classification system for CHD types has not been established; future research should include information that aids in such classification.

## Data Availability

The datasets presented in this study can be found in online repositories. The names of the repository/repositories and accession number(s) can be found in the article/Supplementary Material.
