# Supplementary material for: Evaluating the global, regional, and national burden of congenital heart disease in infants younger than 1 year: a 1990–2021 systematic analysis for the GBD study 2021
Source: Front Pediatr. 2025 Mar 20;13:1467914. doi: 10.3389/fped.2025.1467914 (PMC11966173; doi:10.3389/fped.2025.1467914)
Supplement: Supplementary file 1 [file Datasheet1.zip › Supplementary-figure-S1-S6.docx]

**Figure S1 Prevalent cases of Congenital Heart Disease in Infants in 204 Countries and Territories**


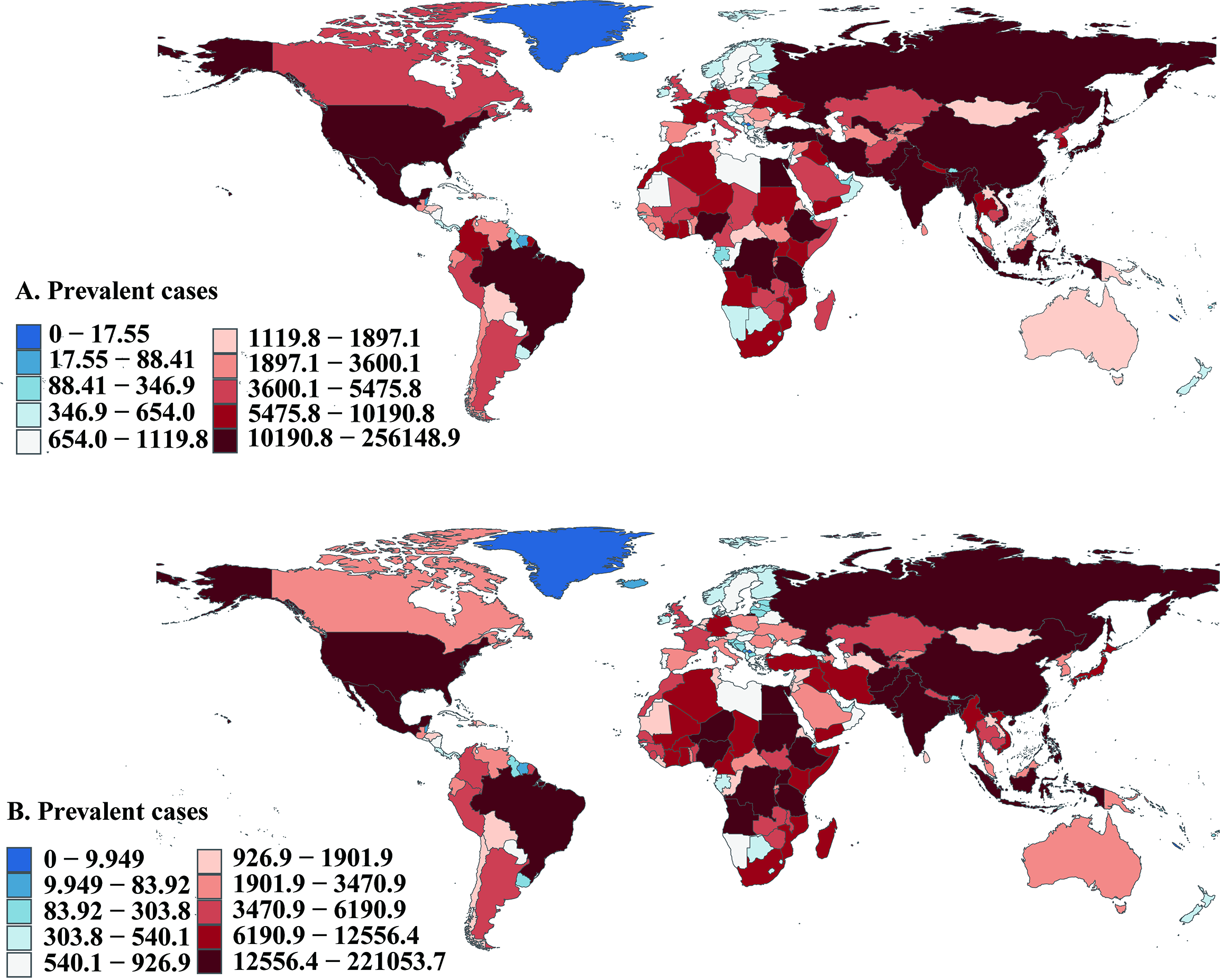


A, Prevalent cases in 1990. B, Prevalent cases in 2021.

**Figure S2 Relationship Between Socio-Demographic Index (SDI) and Prevalence Rate of Congenital Heart Disease in 2021 at the national level.**


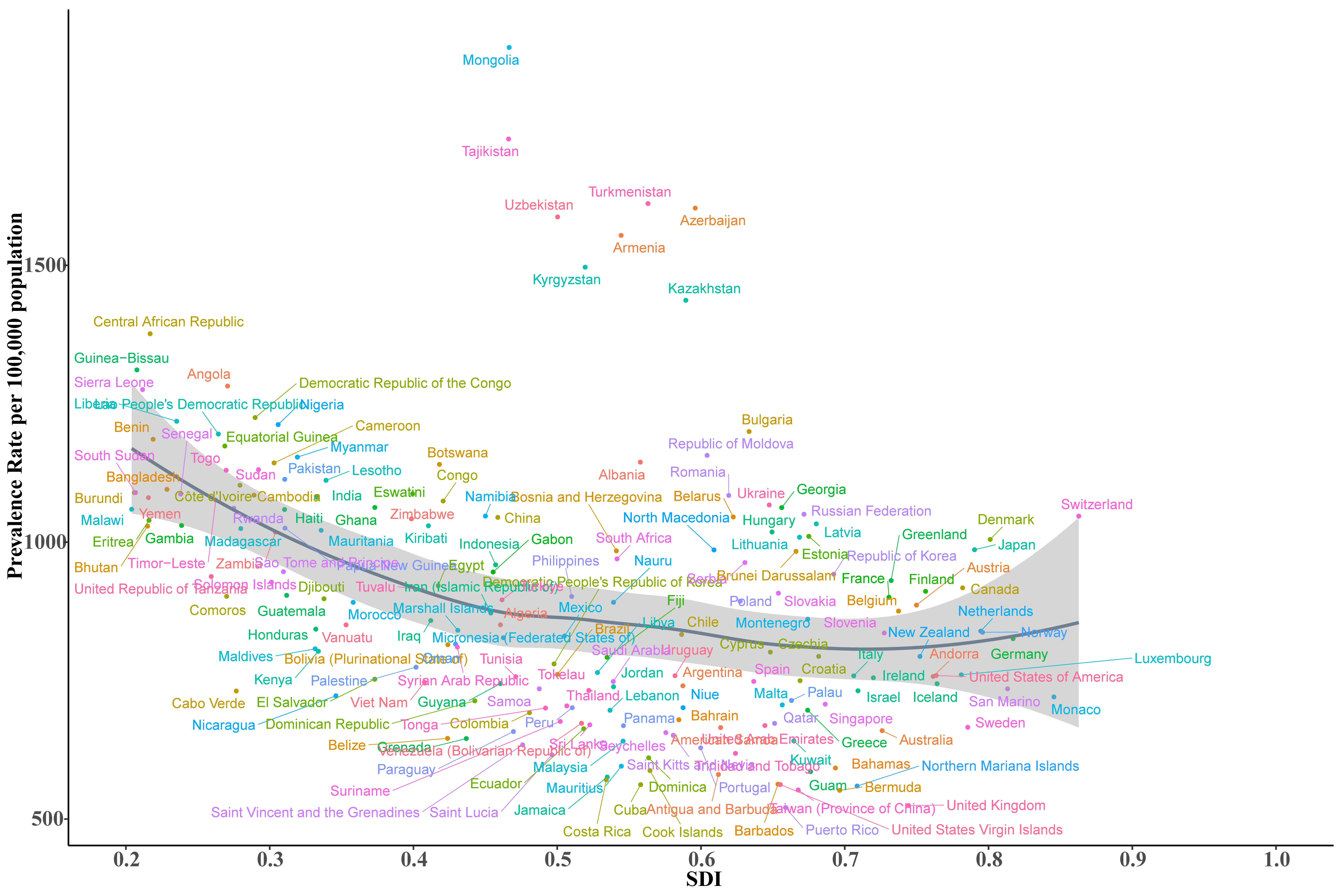


**Figure S3 Deaths Cases of Congenital Heart Disease in Infants in 204 Countries and Territories**

**
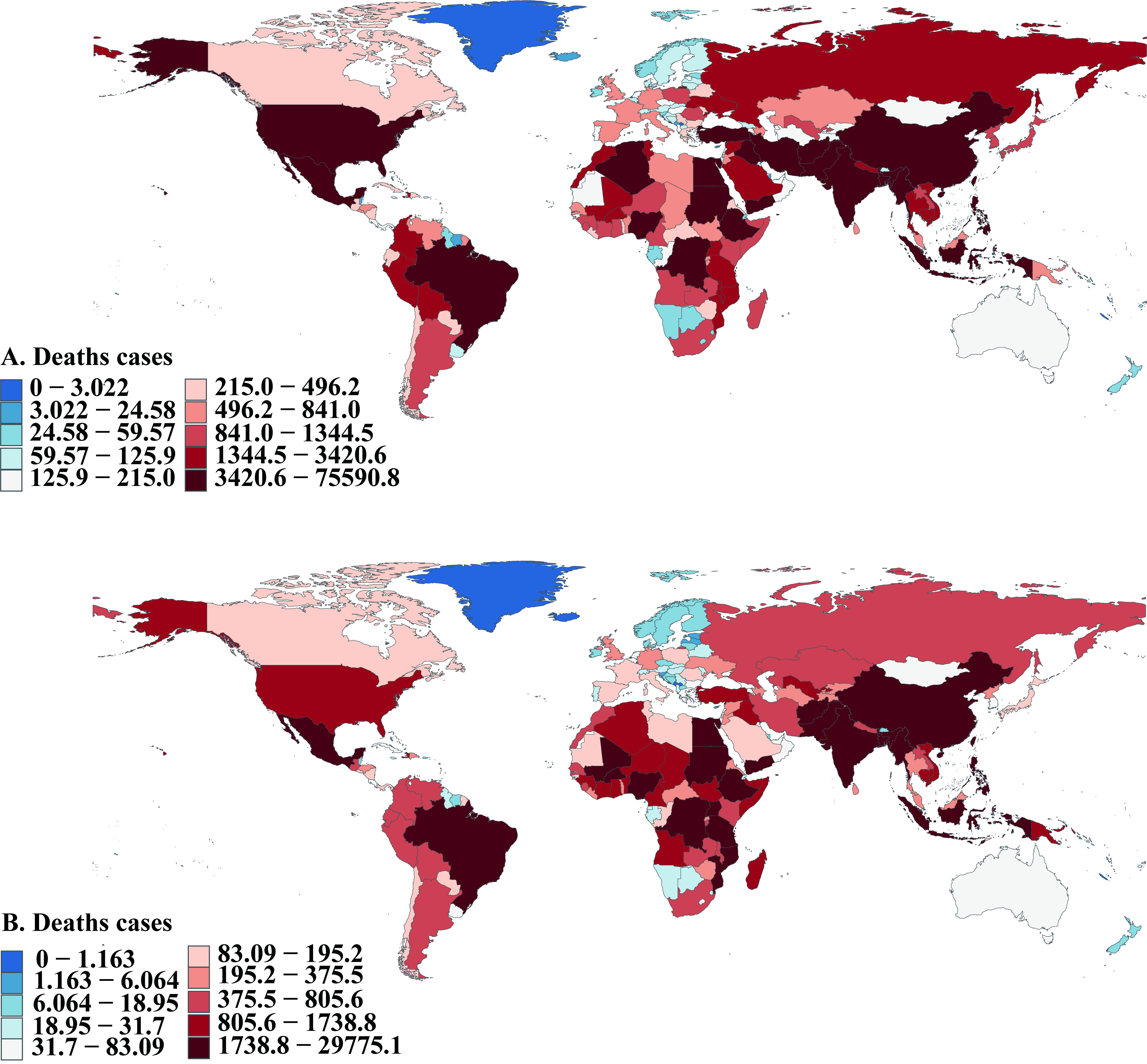
**

A, Deaths cases in 1990. B, Deaths cases in 2021.

**Figure S4 EAPC of Prevalence, Mortality, and Disability-Adjusted Life Years (DALYs) Associated with Congenital Heart Disease in Infants Across 204 Countries and Territories**


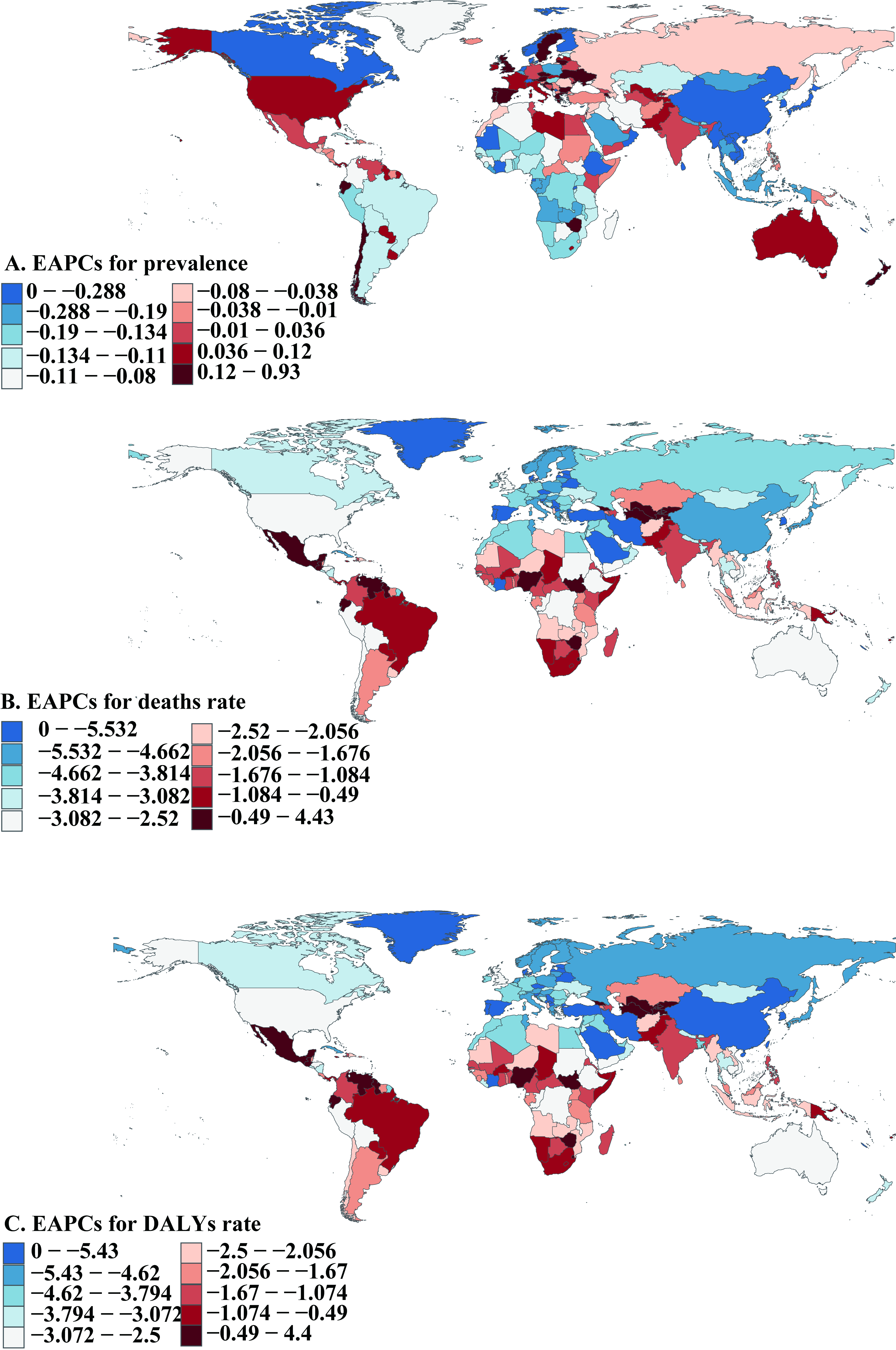


A, EAPCs for prevalence. B, EAPCs for mortality. C, EAPCs for DALYs rate. EAPC, estimated annual percentage change. DALYs, Disability-Adjusted Life Years.

**Figure S5 Disability-Adjusted Life-Years (DALYs) Rates for Congenital Heart Disease in Infants From 1990 to 2021**


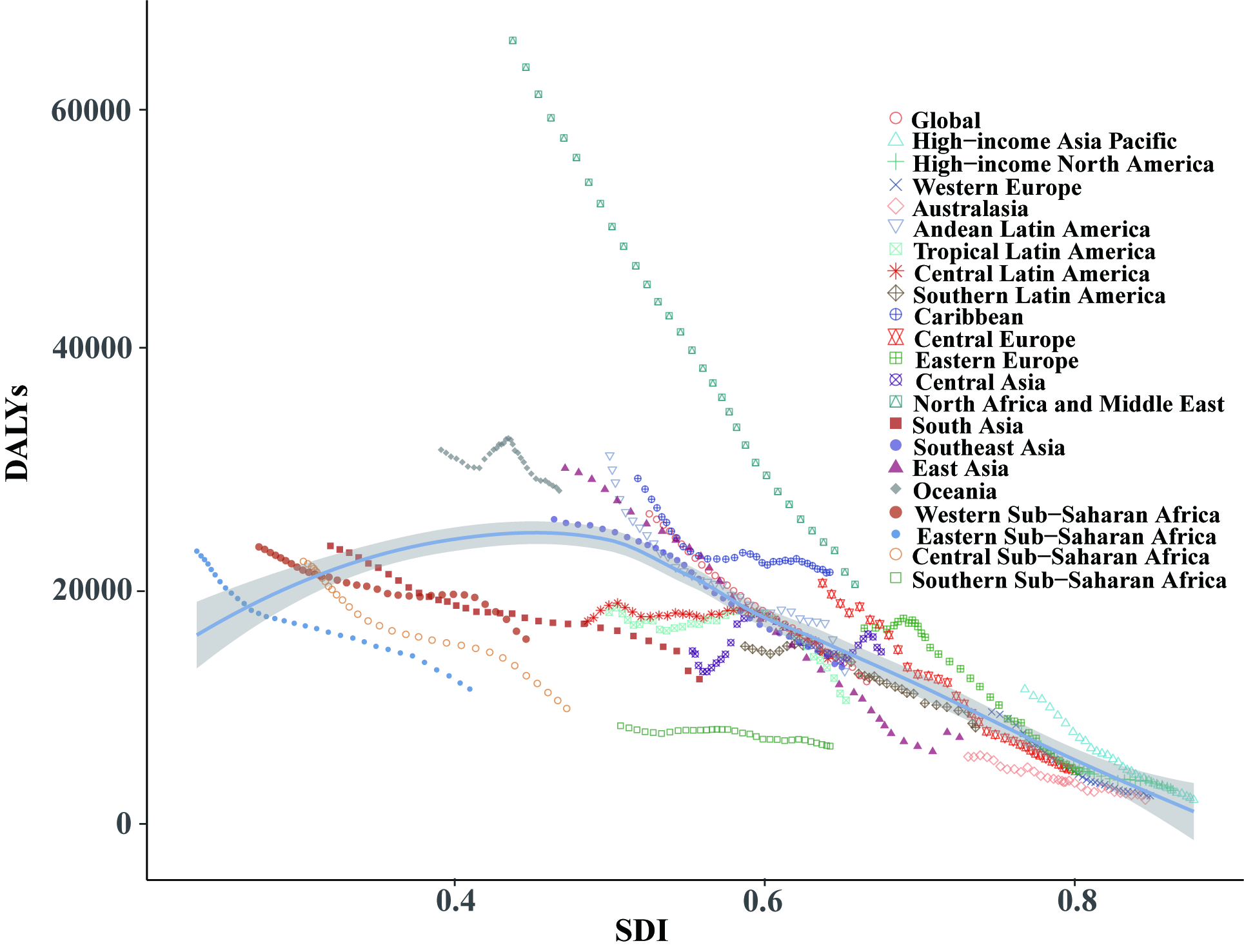


**Figure S6 Disability-Adjusted Life-Years (DALYs) Rates of Congenital Heart Disease in Infants in 204 Countries and Territories**


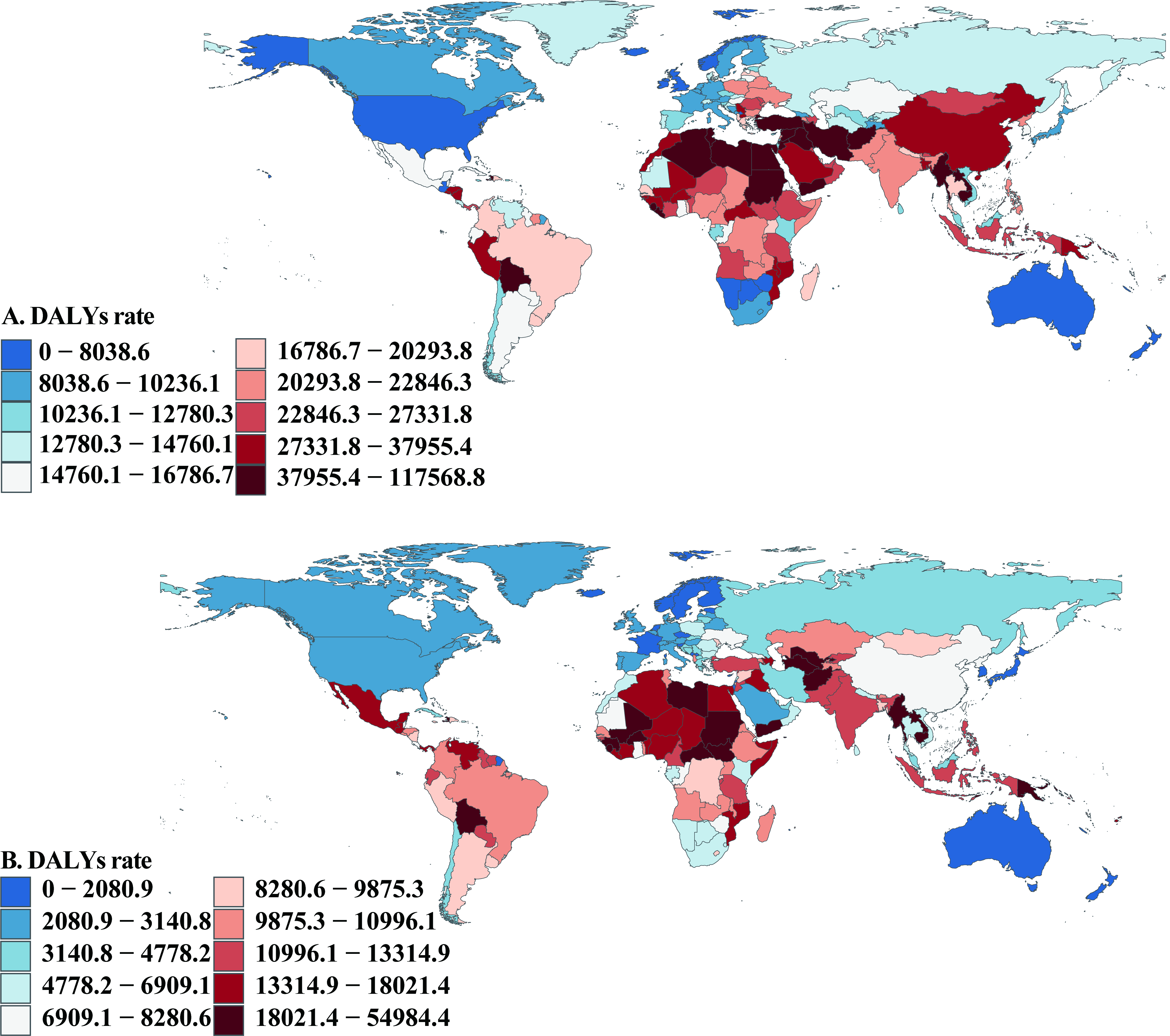


A, DALYs rate in 1990. B, DALYs rate in 2021.
