# Supplementary material for: Evaluating the global, regional, and national burden of congenital heart disease in infants younger than 1 year: a 1990–2021 systematic analysis for the GBD study 2021
Source: Front Pediatr. 2025 Mar 20;13:1467914. doi: 10.3389/fped.2025.1467914 (PMC11966173; doi:10.3389/fped.2025.1467914)

**Figure S7 Trends in Disability-Adjusted Life-Years (DALYs) Rate for Congenital Heart Disease in Infants from 1900 to 2021 Across 204 Countries and Territories**


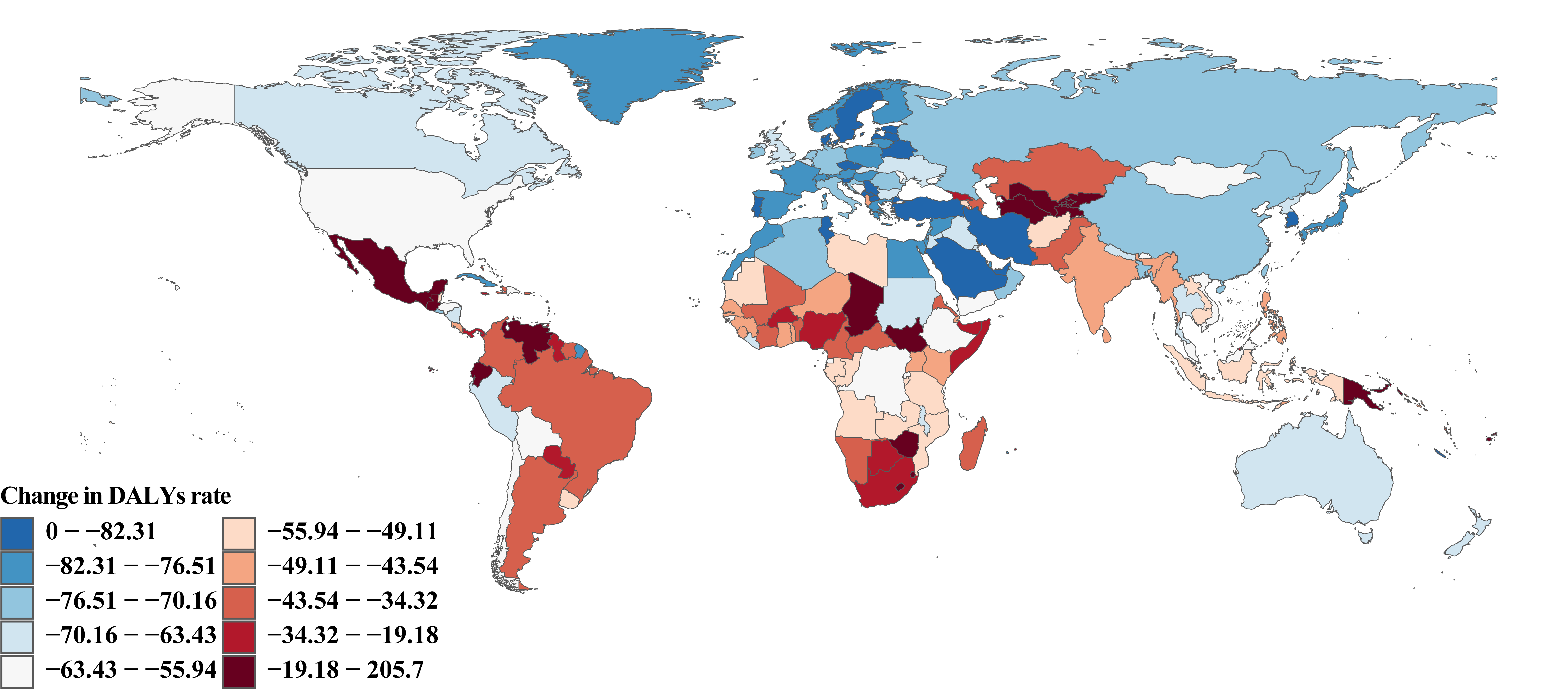


**Figure S8 Prevalence for Congenital Heart Disease in Infants in 2021 Across 204 Countries and Territories**


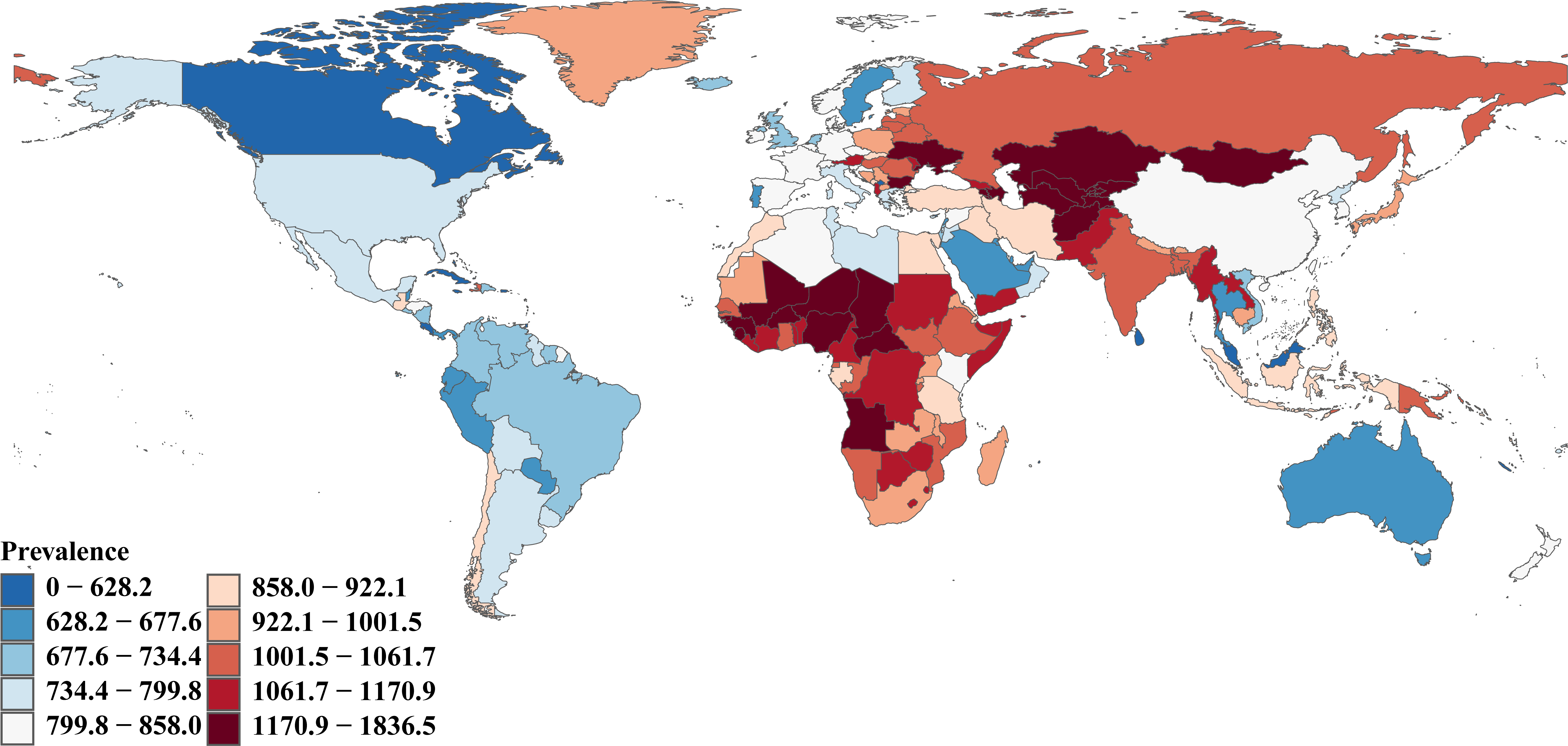


**Figure S9 Mortality for Congenital Heart Disease in Infants Across 204 Countries and Territories**


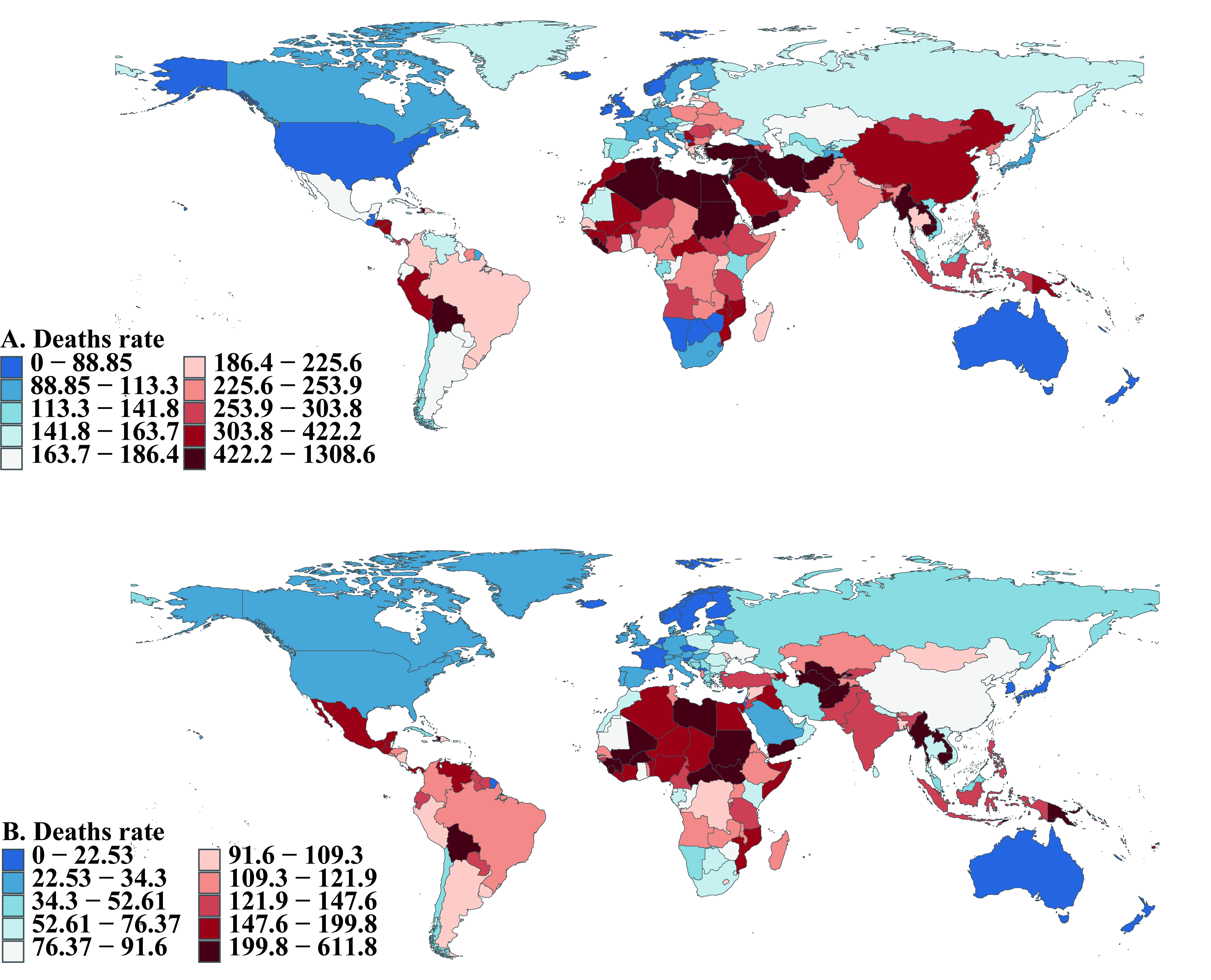


A, **Mortality** in 1990. B, **Mortality** in 2021.

**Figure S10 Trends in Mortality for Congenital Heart Disease in Infants from 1900 to 2021 Across 204 Countries and Territories**


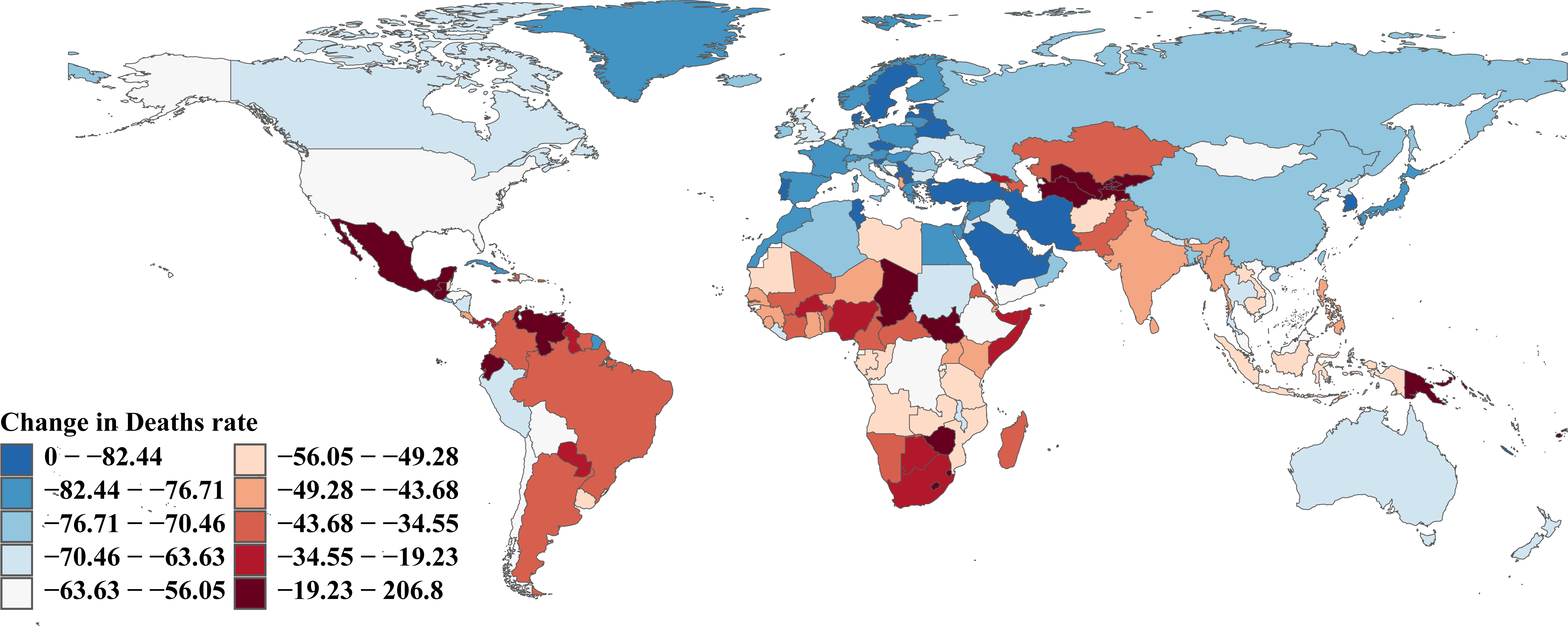


**Figure S11 Deaths Rate for Congenital Heart Disease in Infants From 1990 to 2021**


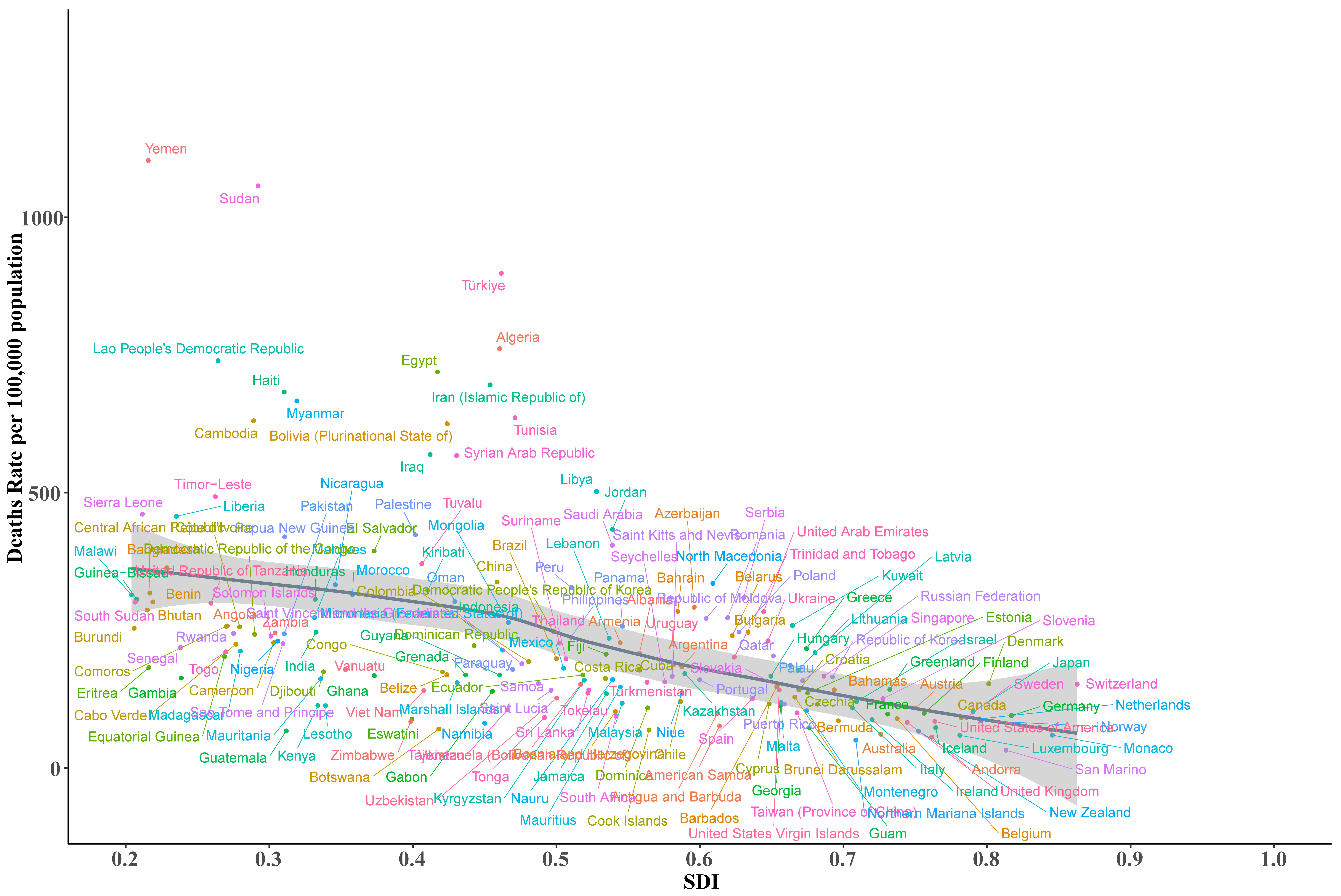


**Figure S12 DALYs Cases for Congenital Heart Disease in Infants Across 204 Countries and Territories**


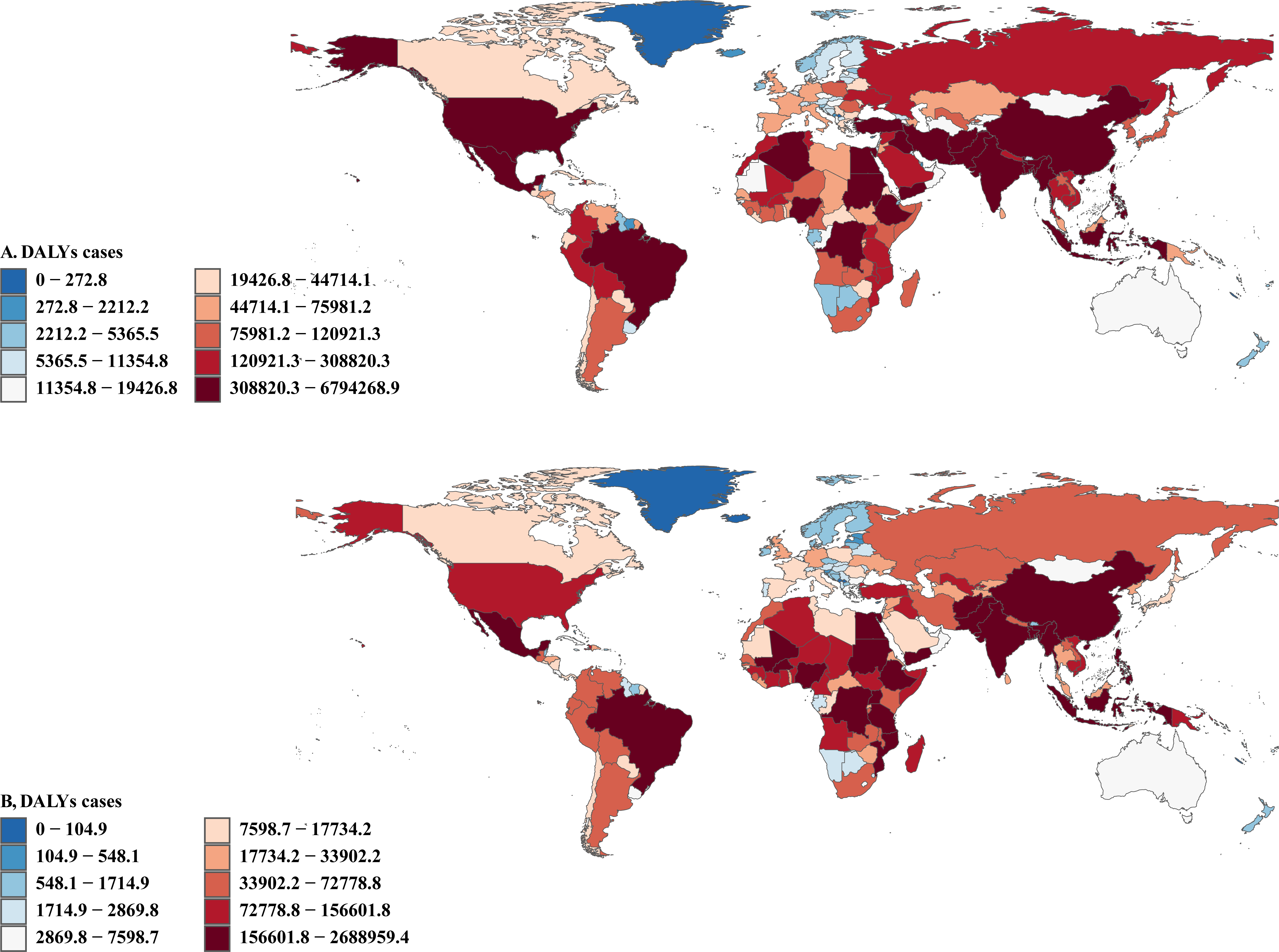


A, DALYs cases in 1990. B, DALYs cases in 2021.

**Figure S13 Disability-Adjusted Life-Years (DALYs) Rates for Congenital Heart Disease in Infants From 1990 to 2021 Across 204 Countries and Territories**


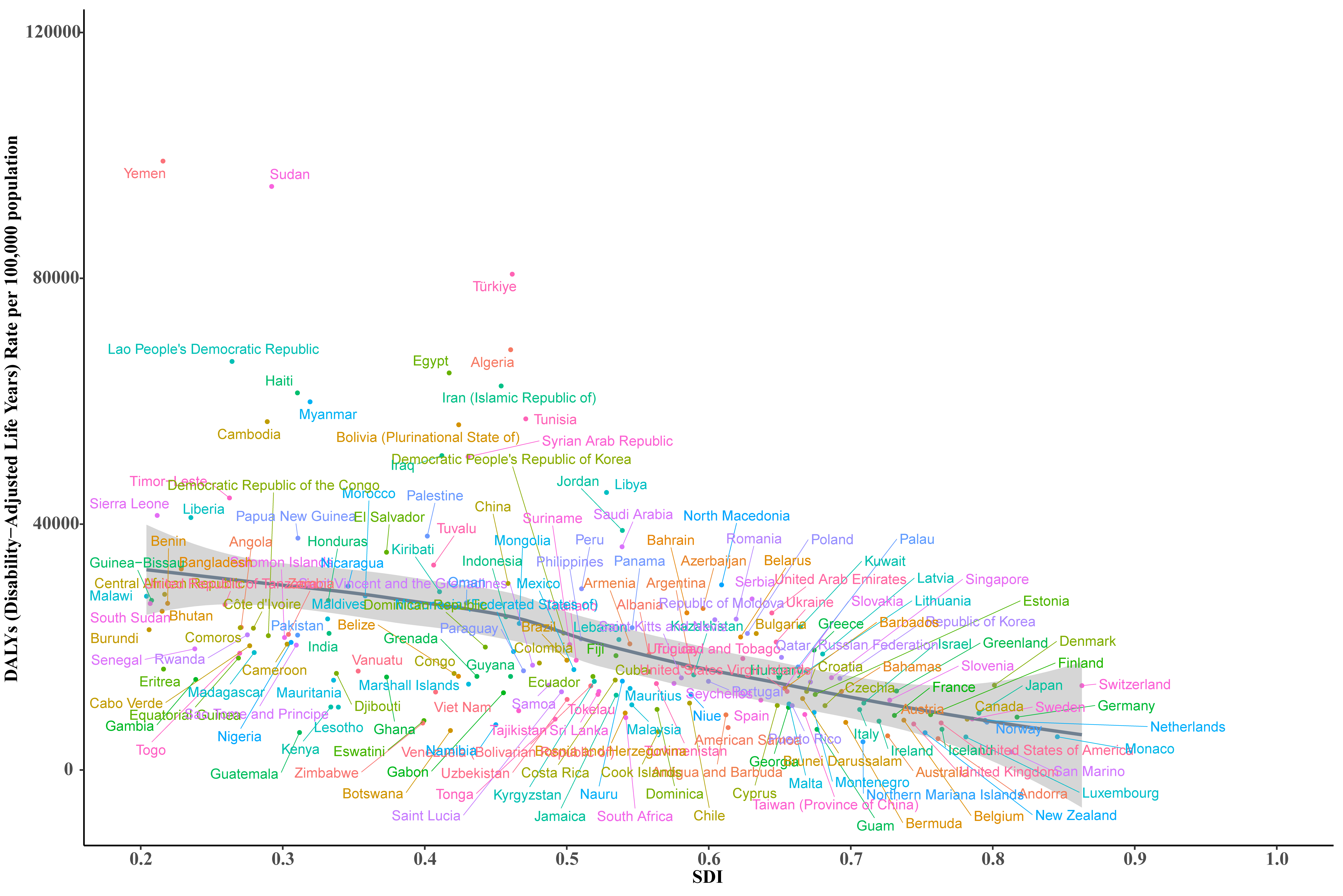

Supplement: Supplementary file 2 [file Datasheet2.zip › Supplementary-figure-S7-S13.docx]
