# Supplementary material for: Evaluating the global, regional, and national burden of congenital heart disease in infants younger than 1 year: a 1990–2021 systematic analysis for the GBD study 2021
Source: Front Pediatr. 2025 Mar 20;13:1467914. doi: 10.3389/fped.2025.1467914 (PMC11966173; doi:10.3389/fped.2025.1467914)
Supplement: Supplementary file 3 [file Table1.docx]

**Table S1 EAPC for Prevalence of Congenital Heart Disease Between 1990 and 2021 at the Global and Regional Level**

| **Location name** | **EAPCs** |
| --- | --- |
| Global | -0.04(-0.07--0.02) |
| High-middle SDI | -0.28(-0.33--0.23) |
| High SDI | -0.12(-0.17--0.07) |
| Middle SDI | -0.24(-0.29--0.20) |
| Low-middle SDI | -0.03(-0.06-0.01) |
| Low SDI | -0.06(-0.08--0.05) |
| Andean Latin America | -0.06(-0.10--0.03) |
| Australasia | 0.08(0.05-0.11) |
| Caribbean | 0.31(0.29-0.33) |
| Central Asia | 0.02(-0.01-0.04) |
| Central Europe | -0.06(-0.10--0.02) |
| Central Latin America | -0.02(-0.06-0.02) |
| Central Sub-Saharan Africa | -0.18(-0.21--0.15) |
| East Asia | -0.66(-0.70--0.62) |
| Eastern Europe | 0.03(0.00-0.05) |
| Eastern Sub-Saharan Africa | -0.17(-0.18--0.16) |
| Global | -0.04(-0.07--0.02) |
| High-income Asia Pacific | -0.32(-0.37--0.26) |
| High-income North America | -0.08(-0.18-0.01) |
| North Africa and Middle East | 0.06(0.04-0.08) |
| Oceania | 0.09(0.07-0.11) |
| South Asia | 0.03(-0.03-0.08) |
| Southeast Asia | -0.17(-0.19--0.14) |
| Southern Latin America | -0.05(-0.13-0.03) |
| Southern Sub-Saharan Africa | -0.05(-0.06--0.04) |
| Tropical Latin America | -0.13(-0.23--0.02) |
| Western Europe | 0.14(0.09-0.18) |
| Western Sub-Saharan Africa | -0.11(-0.12--0.10) |

Data in parentheses are 95% CIs. Abbreviations: EAPC, estimated annual percentage change; SDI, Sociodemographic Index.

**Table S2 Gender Differences in the Prevalence of Congenital Heart Disease Between 1990 and 2021 at the Global and Regional Level**

| **Location name** | **1990-Male** | **1990-Female** | **2021-Male** | **2021-Female** |
| --- | --- | --- | --- | --- |
| Global | 986.84(854.59-1144.10) | 1015.21(869.83-1187.39) | 963.71(833.56-1120.03) | 996.51(857.35-1163.13) |
| Low SDI | 1098.98(943.15-1287.07) | 1220.57(1043.63-1444.33) | 1070.77(916.10-1252.68) | 1186.10(1012.60-1400.05) |
| Middle SDI | 961.85(833.90-1114.41) | 941.15(807.17-1105.96) | 878.02(763.49-1012.46) | 857.75(743.28-998.38) |
| High-middle SDI | 949.40(809.60-1107.12) | 933.70(795.43-1111.07) | 864.77(744.16-1006.17) | 840.66(722.67-981.91) |
| High SDI | 798.66(713.82-894.84) | 797.05(710.09-905.48) | 793.85(703.03-898.53) | 773.69(682.96-879.60) |
| Low-middle SDI | 1035.03(891.48-1207.84) | 1093.93(935.33-1283.71) | 1016.89(876.73-1188.70) | 1047.07(896.60-1236.93) |

Data in parentheses are 95% UI. SDI, Socio-demographic Index. UI, uncertainty interval.

**Table S3 EAPC for Mortality of Congenital Heart Disease Between 1990 and 2021 at the Global and Regional Level**

| **Location name** | **EAPCs** |
| --- | --- |
| Global | -2.35(-2.42--2.28) |
| High SDI | -4.29(-4.46--4.12) |
| High-middle SDI | -5.09(-5.34--4.85) |
| Middle SDI | -3.08(-3.18--2.98) |
| Low-middle SDI | -1.97(-2.11--1.83) |
| Low SDI | -1.57(-1.66--1.47) |
| Andean Latin America | -2.20(-2.38--2.03) |
| Australasia | -3.11(-3.29--2.92) |
| Caribbean | -0.78(-0.95--0.60) |
| Central Asia | 0.24(-0.12-0.60) |
| Central Europe | -4.99(-5.16--4.82) |
| Central Latin America | -0.48(-0.68--0.29) |
| Central Sub-Saharan Africa | -2.39(-2.61--2.17) |
| East Asia | -5.38(-5.74--5.02) |
| Eastern Europe | -4.72(-5.28--4.16) |
| Eastern Sub-Saharan Africa | -2.01(-2.12--1.90) |
| Global | -2.35(-2.42--2.28) |
| High-income Asia Pacific | -5.27(-5.39--5.16) |
| High-income North America | -2.92(-3.10--2.74) |
| North Africa and Middle East | -3.64(-3.73--3.55) |
| Oceania | -0.26(-0.40--0.12) |
| South Asia | -1.64(-1.80--1.49) |
| Southeast Asia | -2.21(-2.34--2.08) |
| Southern Latin America | -1.85(-2.09--1.62) |
| Southern Sub-Saharan Africa | -0.63(-0.75--0.52) |
| Tropical Latin America | -1.07(-1.46--0.68) |
| Western Europe | -4.53(-4.75--4.31) |
| Western Sub-Saharan Africa | -0.98(-1.10--0.87) |

Data in parentheses are 95% CIs. Abbreviations: EAPC, estimated annual percentage change; SDI, Sociodemographic Index.

**Table S4 DALYs of Congenital Heart Disease Between 1990 and 2021 at the Global and Regional Level**

|  | **the number of DALYs** | | |  | **DALYs per 100,000 population** | | |
| --- | --- | --- | --- | --- | --- | --- | --- |
| Location | **1990** | **2021** | **Percent change,1990-2021(%)** |  | **1990** | **2021** | **Percent change,1990-2021(%)** |
| Global | 33278578.86(19684030.06-42527804.16) | 15158581.33(12507441.29-18793629.80) | -54.45(-65.07--23.68) |  | 26050.26(15408.53-33290.49) | 11964.67(9872.13-14833.82) | -54.07(-64.78--23.04) |
| **SDI** |  |  |  |  |  |  |  |
| High SDI | 1339554.73(1154810.59-1463030.35) | 282472.37(226879.08-345814.32) | -78.91(-83.34--72.75) |  | 10856.86(9359.54-11857.61) | 2753.19(2211.33-3370.57) | -74.64(-79.96--67.23) |
| High middle | 4779646.65(3391625.10-5895052.54) | 757449.81(613806.90-913308.03) | -84.15(-88.41--74.66) |  | 26497.55(18802.60-32681.17) | 6362.96(5156.29-7672.25) | -75.99(-82.43--61.60) |
| Middle SDI | 10751397.42(6695309.19-14146117.07) | 3132988.70(2572771.87-3869215.11) | -70.86(-78.94--43.80) |  | 26657.51(16600.66-35074.53) | 9820.26(8064.28-12127.94) | -63.16(-73.38--28.95) |
| Low middle | 10490775.92(5971893.45-13856344.71) | 5278868.50(4112873.42-6669834.36) | -49.68(-63.58-1.87) |  | 28757.00(16369.97-37982.59) | 13897.72(10828.00-17559.73) | -51.67(-65.02--2.17) |
| Low SDI | 5888843.07(2328437.65-8859371.92) | 5689689.21(4016523.89-7743144.24) | -3.38(-27.18-77.44) |  | 28788.93(11383.09-43311.03) | 16471.95(11628.05-22416.81) | -42.78(-56.88-5.08) |
| **Regions** |  |  |  |  |  |  |  |
| Andean Latin America | 346489.896(193574.611-454020.510) | 157031.353(115626.167-203326.484) | -54.68(-69.81--7.90) |  | 30970.786(17302.548-40582.344) | 12847.229(9459.740-16634.779) | -58.52(-72.37--15.70) |
| Australasia | 17591.386(16008.622-19661.360) | 7156.835(5151.536-9089.080) | -59.32(-70.81--48.15) |  | 5650.099(5141.738-6314.945) | 2022.734(1455.977-2568.843) | -64.20(-74.31--54.37) |
| Caribbean | 252148.284(198224.128-309720.756) | 163841.964(107253.308-248732.735) | -35.02(-54.89-4.38) |  | 29025.448(22818.097-35652.765) | 21141.132(13839.289-32094.900) | -27.16(-49.44-17.00) |
| Central Asia | 278484.788(242194.693-314523.289) | 292228.108(227925.489-365071.120) | 4.94(-16.47-30.36) |  | 14532.207(12638.476-16412.808) | 14459.855(11278.071-18064.229) | -0.50(-20.80-23.61) |
| Central Europe | 347895.632(295393.954-388758.776) | 48624.689(38934.035-58148.237) | -86.02(-89.73--82.66) |  | 20229.498(17176.621-22605.616) | 4615.148(3695.373-5519.064) | -77.19(-83.24--71.69) |
| Central Latin America | 820237.158(718203.638-942239.356) | 538353.364(404626.686-696199.058) | -34.37(-51.98--12.08) |  | 17034.399(14915.403-19568.098) | 13899.455(10446.838-17974.788) | -18.40(-40.30-9.31) |
| Central Sub-Saharan Africa | 524902.747(181539.258-975471.532) | 416596.170(260504.971-693254.257) | -20.63(-43.73-72.81) |  | 22054.399(7627.583-40985.570) | 9698.977(6064.942-16139.988) | -56.02(-68.82--4.25) |
| East Asia | 6939349.410(4296775.764-9800609.039) | 868015.628(655714.680-1144021.383) | -87.49(-92.00--76.01) |  | 29875.551(18498.643-42193.955) | 7268.409(5490.688-9579.569) | -75.67(-84.44--53.33) |
| Eastern Europe | 502409.753(445112.494-596077.290) | 81010.237(62809.021-98649.817) | -83.88(-88.61--79.00) |  | 16433.355(14559.215-19497.133) | 4459.094(3457.234-5430.040) | -72.87(-80.83--64.66) |
| Eastern Sub-Saharan Africa | 1892214.675(562907.314-3756380.754) | 1494400.753(924205.062-2672837.343) | -21.02(-44.86-83.96) |  | 22917.397(6817.604-45495.085) | 11332.345(7008.435-20268.669) | -50.55(-65.47-15.18) |
| High-income Asia Pacific | 220371.320(174985.024-253869.797) | 23934.235(18335.429-32593.433) | -89.14(-91.33--82.38) |  | 11303.231(8975.288-13021.426) | 2010.743(1540.381-2738.213) | -82.21(-85.79--71.13) |
| High-income North America | 346374.093(295524.206-379761.572) | 113542.940(93704.722-141585.347) | -67.22(-73.89--55.99) |  | 7709.993(6578.117-8453.169) | 2824.139(2330.706-3521.634) | -63.37(-70.83--50.82) |
| North Africa and Middle East | 6908496.425(3126508.978-9622820.292) | 2380018.181(1887675.896-2979651.331) | -65.55(-75.08--34.36) |  | 65821.110(29788.000-91681.992) | 20128.684(15964.765-25200.001) | -69.42(-77.88--41.73) |
| Oceania | 67387.476(22307.036-100207.968) | 115434.395(44612.437-175682.111) | 71.30(29.16-141.17) |  | 31431.610(10404.694-46740.106) | 27994.290(10819.076-42605.118) | -10.94(-32.84-25.39) |
| South Asia | 7557052.731(5120645.659-10013121.882) | 3751572.083(2592766.378-5449500.607) | -50.36(-66.21--0.03) |  | 23354.908(15825.244-30945.336) | 12164.111(8406.795-17669.480) | -47.92(-64.55-4.89) |
| Southeast Asia | 3038651.986(1480725.066-4117271.739) | 1459642.262(1181465.933-1823589.204) | -51.96(-64.51-0.29) |  | 25593.222(12471.493-34677.960) | 13181.182(10669.133-16467.775) | -48.50(-61.95-7.53) |
| Southern Latin America | 153468.285(127002.164-184490.068) | 62284.920(49203.113-78394.060) | -59.42(-69.72--46.77) |  | 14924.863(12351.020-17941.746) | 8109.028(6405.875-10206.317) | -45.67(-59.46--28.74) |
| Southern Sub-Saharan Africa | 127852.014(103832.094-167163.221) | 103988.449(70542.046-147004.514) | -18.66(-43.94-16.14) |  | 8253.902(6703.218-10791.765) | 6534.793(4432.970-9237.989) | -20.83(-45.43-13.06) |
| Tropical Latin America | 580550.542(486979.792-681310.177) | 354676.264(280268.084-435592.187) | -38.91(-54.62--19.31) |  | 17792.311(14924.620-20880.323) | 10386.729(8207.678-12756.360) | -41.62(-56.63--22.90) |
| Western Europe | 431365.172(371057.975-473358.735) | 96604.213(75006.237-117948.362) | -77.61(-83.17--70.76) |  | 9417.607(8100.975-10334.415) | 2366.254(1837.226-2889.064) | -74.87(-81.12--67.19) |
| Western Sub-Saharan Africa | 1925285.083(572780.395-2739773.654) | 2629624.281(1578973.905-3544155.976) | 36.58(4.16-196.91) |  | 23272.821(6923.762-33118.348) | 15527.290(9323.456-20927.377) | -33.28(-49.12-45.04) |

Data in parentheses are 95% UI. SDI, Socio-demographic Index. UI, uncertainty interval. DALYs, Disability-Adjusted Life Years.

**Table S5 EAPC for DALYs of Congenital Heart Disease Between 1990 and 2021 at the Global and Regional Level**

| **Location name** | **EAPCs** |
| --- | --- |
| Global | -2.34(-2.41--2.27) |
| High-middle SDI | -5.07(-5.31--4.83) |
| High SDI | -4.25(-4.42--4.08) |
| Low-middle SDI | -1.97(-2.10--1.83) |
| Low SDI | -1.56(-1.65--1.47) |
| Andean Latin America | -2.20(-2.37--2.02) |
| Australasia | -3.06(-3.25--2.88) |
| Caribbean | -0.77(-0.95--0.60) |
| Central Asia | 0.24(-0.12-0.60) |
| Central Europe | -4.95(-5.12--4.79) |
| Central Latin America | -0.48(-0.67--0.29) |
| Central Sub-Saharan Africa | -2.38(-2.60--2.16) |
| East Asia | -5.36(-5.71--5.00) |
| Eastern Europe | -4.69(-5.25--4.14) |
| Eastern Sub-Saharan Africa | -2.00(-2.11--1.90) |
| Global | -2.34(-2.41--2.27) |
| High-income Asia Pacific | -5.21(-5.33--5.09) |
| High-income North America | -2.89(-3.07--2.71) |
| North Africa and Middle East | -3.63(-3.72--3.54) |
| Oceania | -0.26(-0.40--0.13) |
| South Asia | -1.64(-1.79--1.48) |
| Southeast Asia | -2.20(-2.33--2.07) |
| Southern Latin America | -1.84(-2.08--1.61) |
| Southern Sub-Saharan Africa | -0.63(-0.75--0.51) |
| Tropical Latin America | -1.07(-1.46--0.67) |
| Western Europe | -4.47(-4.69--4.25) |
| Western Sub-Saharan Africa | -0.98(-1.10--0.86) |

Data in parentheses are 95% CIs. Abbreviations: EAPC, estimated annual percentage change; SDI, Sociodemographic Index. DALYs, Disability-Adjusted Life Years.

**Table S6 Socio-Demographic Index Scores in 2021 at the Global and Regional Level**

| Location name | SDI |
| --- | --- |
| High-income Asia Pacific | 0.876767 |
| High-income North America | 0.863465474 |
| Western Europe | 0.848726316 |
| Australasia | 0.845514063 |
| Eastern Europe | 0.802851009 |
| Central Europe | 0.796244448 |
| Southern Latin America | 0.735984717 |
| East Asia | 0.725704902 |
| Central Asia | 0.675163978 |
| Global | 0.665820975 |
| North Africa and Middle East | 0.658224715 |
| Tropical Latin America | 0.652442394 |
| Andean Latin America | 0.651602456 |
| Southeast Asia | 0.649777295 |
| Southern Sub-Saharan Africa | 0.642200282 |
| Caribbean | 0.642003055 |
| Central Latin America | 0.6406851 |
| South Asia | 0.557864657 |
| Central Sub-Saharan Africa | 0.472255651 |
| Oceania | 0.467445126 |
| Western Sub-Saharan Africa | 0.446022979 |
| Eastern Sub-Saharan Africa | 0.409720983 |

SDI, Socio-Demographic Index.

**Table S7 EAPC for Mortality of Congenital Heart Disease Between 1990 and 2021 at the National Level**

|  |  |
| --- | --- |
| **Location name** | **EAPCs** |
| Croatia | 0.93(0.72-1.13) |
| United Kingdom | 0.86(0.77-0.96) |
| Austria | 0.49(0.30-0.68) |
| Bulgaria | 0.43(0.31-0.55) |
| Spain | 0.43(0.29-0.56) |
| Greece | 0.42(0.28-0.55) |
| Ukraine | 0.42(0.35-0.48) |
| Portugal | 0.39(0.30-0.49) |
| Malta | 0.38(0.26-0.51) |
| Sweden | 0.36(0.22-0.50) |
| Czechia | 0.31(0.16-0.45) |
| New Zealand | 0.27(0.22-0.32) |
| Zimbabwe | 0.19(0.15-0.22) |
| Chile | 0.17(0.12-0.22) |
| Montenegro | 0.16(0.11-0.21) |
| Kuwait | 0.15(0.13-0.17) |
| Lithuania | 0.15(0.09-0.20) |
| Ecuador | 0.14(0.03-0.26) |
| Armenia | 0.14(0.09-0.19) |
| Libya | 0.12(0.10-0.14) |
| Italy | 0.12(0.02-0.22) |
| Tajikistan | 0.12(0.09-0.14) |
| San Marino | 0.11(0.08-0.14) |
| France | 0.10(0.02-0.17) |
| Latvia | 0.09(0.06-0.12) |
| Pakistan | 0.09(0.04-0.13) |
| Lesotho | 0.09(0.06-0.11) |
| Uruguay | 0.09(0.05-0.12) |
| Dominica | 0.08(0.07-0.10) |
| Jamaica | 0.07(0.05-0.09) |
| United States of America | 0.07(-0.03-0.17) |
| Ireland | 0.06(-0.08-0.21) |
| Slovakia | 0.06(0.04-0.09) |
| Paraguay | 0.06(0.04-0.08) |
| Monaco | 0.05(0.02-0.09) |
| Uzbekistan | 0.05(0.02-0.08) |
| Australia | 0.05(0.02-0.08) |
| Guyana | 0.04(0.02-0.07) |
| Palestine | 0.04(0.01-0.07) |
| Saint Vincent and the Grenadines | 0.04(0.02-0.06) |
| Panama | 0.04(0.00-0.07) |
| Yemen | 0.03(-0.00-0.06) |
| Turkmenistan | 0.03(0.01-0.05) |
| Venezuela (Bolivarian Republic of) | 0.03(-0.01-0.06) |
| United Arab Emirates | 0.03(-0.01-0.06) |
| Kenya | 0.03(0.01-0.04) |
| India | 0.02(-0.06-0.10) |
| Tunisia | 0.02(-0.00-0.04) |
| Germany | 0.02(-0.27-0.30) |
| Grenada | 0.01(-0.00-0.03) |
| Republic of Moldova | 0.01(-0.02-0.05) |
| Barbados | 0.01(-0.01-0.03) |
| Egypt | 0.00(-0.02-0.03) |
| Belarus | 0.00(-0.02-0.03) |
| United States Virgin Islands | 0.00(-0.01-0.01) |
| Mexico | -0.00(-0.08-0.08) |
| Bosnia and Herzegovina | -0.00(-0.03-0.03) |
| Georgia | -0.00(-0.05-0.04) |
| Dominican Republic | -0.01(-0.04-0.02) |
| Iceland | -0.01(-0.06-0.04) |
| Sudan | -0.01(-0.02--0.00) |
| Belize | -0.01(-0.04-0.02) |
| Andorra | -0.01(-0.05-0.03) |
| Serbia | -0.01(-0.03-0.01) |
| Haiti | -0.01(-0.03-0.01) |
| Honduras | -0.02(-0.03-0.00) |
| Papua New Guinea | -0.02(-0.04-0.00) |
| Suriname | -0.02(-0.05-0.01) |
| Afghanistan | -0.02(-0.07-0.03) |
| Vanuatu | -0.02(-0.04--0.00) |
| Slovenia | -0.02(-0.05-0.00) |
| Northern Mariana Islands | -0.02(-0.05--0.00) |
| North Macedonia | -0.02(-0.06-0.01) |
| Central African Republic | -0.02(-0.04--0.01) |
| Philippines | -0.03(-0.04--0.01) |
| Turkey | -0.03(-0.11-0.06) |
| Nicaragua | -0.03(-0.06-0.00) |
| Costa Rica | -0.03(-0.06--0.01) |
| Bahamas | -0.03(-0.06--0.00) |
| American Samoa | -0.03(-0.05--0.02) |
| Somalia | -0.03(-0.05--0.02) |
| Brunei Darussalam | -0.04(-0.06--0.01) |
| Syrian Arab Republic | -0.04(-0.08--0.00) |
| Fiji | -0.04(-0.07--0.02) |
| Romania | -0.04(-0.06--0.03) |
| Kyrgyzstan | -0.04(-0.07--0.02) |
| Tonga | -0.04(-0.06--0.03) |
| Azerbaijan | -0.05(-0.07--0.02) |
| Eswatini | -0.05(-0.07--0.02) |
| Trinidad and Tobago | -0.05(-0.07--0.03) |
| Saint Lucia | -0.05(-0.07--0.03) |
| Morocco | -0.05(-0.08--0.02) |
| Russian Federation | -0.05(-0.08--0.03) |
| Guam | -0.06(-0.10--0.01) |
| Qatar | -0.06(-0.09--0.02) |
| Antigua and Barbuda | -0.06(-0.08--0.03) |
| Puerto Rico | -0.06(-0.09--0.04) |
| Guatemala | -0.07(-0.09--0.04) |
| Israel | -0.07(-0.17-0.03) |
| Jordan | -0.07(-0.10--0.05) |
| Solomon Islands | -0.08(-0.08--0.07) |
| Marshall Islands | -0.08(-0.09--0.06) |
| Iraq | -0.08(-0.12--0.04) |
| Iran (Islamic Republic of) | -0.08(-0.11--0.06) |
| Algeria | -0.08(-0.09--0.07) |
| Bermuda | -0.09(-0.10--0.07) |
| Nauru | -0.09(-0.13--0.05) |
| Kiribati | -0.09(-0.10--0.07) |
| Taiwan (Province of China) | -0.09(-0.15--0.03) |
| Luxembourg | -0.09(-0.11--0.07) |
| Chad | -0.09(-0.11--0.07) |
| Botswana | -0.09(-0.13--0.06) |
| Madagascar | -0.09(-0.11--0.08) |
| Colombia | -0.09(-0.11--0.08) |
| South Sudan | -0.10(-0.11--0.08) |
| Mauritius | -0.10(-0.14--0.05) |
| Albania | -0.10(-0.15--0.05) |
| Burkina Faso | -0.10(-0.12--0.08) |
| El Salvador | -0.10(-0.13--0.08) |
| Guinea | -0.10(-0.13--0.08) |
| Greenland | -0.10(-0.15--0.06) |
| Mozambique | -0.11(-0.13--0.08) |
| Senegal | -0.11(-0.13--0.08) |
| Ghana | -0.11(-0.13--0.08) |
| Bahrain | -0.11(-0.14--0.08) |
| Niue | -0.11(-0.13--0.09) |
| Palau | -0.11(-0.14--0.09) |
| Samoa | -0.11(-0.14--0.09) |
| United Republic of Tanzania | -0.11(-0.14--0.09) |
| Nigeria | -0.12(-0.13--0.10) |
| Burundi | -0.12(-0.14--0.10) |
| Sierra Leone | -0.12(-0.14--0.10) |
| Bolivia (Plurinational State of) | -0.12(-0.14--0.10) |
| Saint Kitts and Nevis | -0.12(-0.14--0.10) |
| Lebanon | -0.12(-0.15--0.10) |
| Democratic People's Republic of Korea | -0.12(-0.16--0.09) |
| Argentina | -0.12(-0.23--0.02) |
| Cuba | -0.13(-0.15--0.11) |
| Gambia | -0.13(-0.15--0.11) |
| Estonia | -0.13(-0.18--0.08) |
| Brazil | -0.13(-0.24--0.02) |
| Kazakhstan | -0.13(-0.17--0.10) |
| Benin | -0.14(-0.15--0.12) |
| Togo | -0.14(-0.16--0.12) |
| Seychelles | -0.15(-0.18--0.11) |
| Cameroon | -0.15(-0.17--0.13) |
| Peru | -0.15(-0.17--0.13) |
| Comoros | -0.15(-0.17--0.14) |
| Uganda | -0.15(-0.19--0.12) |
| Micronesia (Federated States of) | -0.16(-0.18--0.14) |
| Guinea-Bissau | -0.16(-0.17--0.14) |
| Eritrea | -0.16(-0.17--0.15) |
| C涔坱e d'Ivoire | -0.16(-0.20--0.13) |
| South Africa | -0.16(-0.18--0.15) |
| Bhutan | -0.17(-0.18--0.15) |
| Democratic Republic of the Congo | -0.17(-0.20--0.13) |
| Djibouti | -0.17(-0.21--0.13) |
| Malawi | -0.17(-0.20--0.15) |
| Hungary | -0.18(-0.30--0.05) |
| Mali | -0.18(-0.21--0.15) |
| Namibia | -0.18(-0.21--0.16) |
| Niger | -0.18(-0.21--0.16) |
| Sao Tome and Principe | -0.19(-0.21--0.17) |
| Tuvalu | -0.19(-0.22--0.17) |
| Cyprus | -0.19(-0.22--0.17) |
| Bangladesh | -0.20(-0.22--0.18) |
| Indonesia | -0.20(-0.23--0.17) |
| Belgium | -0.21(-0.42--0.00) |
| Cabo Verde | -0.22(-0.24--0.19) |
| Tokelau | -0.22(-0.23--0.21) |
| Zambia | -0.22(-0.27--0.18) |
| Nepal | -0.22(-0.27--0.17) |
| Timor-Leste | -0.22(-0.25--0.20) |
| Congo | -0.23(-0.25--0.20) |
| Mongolia | -0.24(-0.29--0.19) |
| Poland | -0.24(-0.41--0.07) |
| Malaysia | -0.25(-0.29--0.21) |
| Gabon | -0.26(-0.28--0.24) |
| Cook Islands | -0.26(-0.30--0.23) |
| Angola | -0.27(-0.30--0.24) |
| Thailand | -0.28(-0.31--0.25) |
| Liberia | -0.28(-0.31--0.26) |
| Saudi Arabia | -0.28(-0.35--0.22) |
| Japan | -0.29(-0.35--0.22) |
| Mauritania | -0.29(-0.31--0.27) |
| Sri Lanka | -0.30(-0.32--0.27) |
| Lao People's Democratic Republic | -0.32(-0.34--0.29) |
| Myanmar | -0.32(-0.36--0.29) |
| Cambodia | -0.32(-0.34--0.30) |
| Viet Nam | -0.33(-0.36--0.30) |
| Norway | -0.34(-0.52--0.17) |
| Rwanda | -0.36(-0.39--0.32) |
| Oman | -0.37(-0.44--0.30) |
| Ethiopia | -0.38(-0.39--0.36) |
| Republic of Korea | -0.42(-0.46--0.37) |
| Maldives | -0.46(-0.51--0.41) |
| Netherlands | -0.49(-0.61--0.37) |
| Singapore | -0.52(-0.58--0.46) |
| Equatorial Guinea | -0.57(-0.61--0.52) |
| Switzerland | -0.60(-0.71--0.49) |
| China | -0.69(-0.73--0.65) |
| Finland | -0.76(-1.01--0.51) |
| Denmark | -0.82(-1.03--0.61) |
| Canada | -1.74(-1.94--1.54) |

Data in parentheses are 95% CIs. Abbreviations: EAPC, estimated annual percentage change.
